# Neurovascular coupling unit dysfunction and dementia: Retinal measurements as tools to move towards population-based evidence

**DOI:** 10.3389/fendo.2022.1014287

**Published:** 2022-11-23

**Authors:** Frank C. T. van der Heide, Thomas T. van Sloten, Nele Willekens, Coen D. A. Stehouwer

**Affiliations:** ^1^ CARIM School for Cardiovascular Diseases, Maastricht University (UM), Maastricht, Netherlands; ^2^ Department of Internal Medicine, Maastricht University Medical Center+ (MUMC+), Maastricht, Netherlands; ^3^ Department of Psychiatry and Neuropsychology, MUMC+, Maastricht, Netherlands; ^4^ School of Mental Health and Neuroscience, MUMC+, Maastricht, Netherlands

**Keywords:** Dementia, mild cognitive impairment, epidemiology, retinal imaging, optical coherence tomography, blood-brain barrier, neurovascular coupling unit, magnetic resonance imaging

## Abstract

Dysfunction of the neurovascular coupling unit may be an important contributor to dementia. The neurovascular coupling unit comprises neuronal structures (e.g. astrocytes) and vascular structures (e.g. endothelial cells) that functionally interact both at the level of the arterioles as well as at the capillary level (blood-brain barrier) to regulate optimal metabolic conditions in the brain. However, it remains unclear how and to what extent dysfunction of the neurovascular coupling unit contributes to the early-stage pathobiology of dementia. Currently, limited data are available on the association between neurovascular coupling unit dysfunction, as quantified by cerebral imaging techniques, and cognitive performance. In particular, there is a lack of population-based human data (defined as studies with a sample size ~n>500). This is an important limitation because population-based studies, in comparison with smaller clinical studies, provide data which is better representative of the general population; are less susceptible to selection bias; and have a larger statistical power to detect small associations. To acquire population-based data, however, alternative imaging techniques than cerebral imaging techniques may be required. Disadvantages of cerebral imaging techniques, which limit use in population-based studies, are that these techniques are relatively expensive, time-consuming, and/or invasive. In this review, we propose that retinal imaging techniques can be used for population-based studies: on the one hand the retina and brain have many anatomical and physiological similarities; and on the other hand retinal imaging techniques are non-invasive, highly accurate, relatively inexpensive, and require relatively short measurement time. To provide support for this concept, we provide an overview on the human (population-based) evidence on the associations of retinal indices of neurodegeneration, microvascular dysfunction, and dysfunction of the neurovascular coupling unit with magnetic resonance imaging (MRI) features of structural brain abnormalities and cognitive performance.

## Introduction

There is an imperative for the development of novel therapeutic strategies that aim to prevent dementia at an early stage (1–3). Dementia can have debilitating consequences on the quality of life, is associated with a high risk of mortality, and has a large burden on healthcare systems (1–3). Globally, approximately ~55 million individuals currently have dementia and a dramatic increase (up to ~90%) is anticipated in the next decades (1, 4). A better understanding of the early pathophysiology of dementia is crucial for development of effective strategies to prevent and/or delay the onset of dementia (3, 5).

Dysfunction of the neurovascular coupling unit is considered an important contributor to the onset of different types of dementia, including Alzheimer’s disease and vascular dementia. The neurovascular coupling unit comprises neuronal structures (e.g. glial cells) and vascular structures (e.g. endothelial cells) in the brain that collaborate in a highly coordinated manner to regulate metabolic circumstances for optimal brain function (6, 7). (**Box 1**) Degeneration of neuronal and vascular structures can lead to dysfunction of the neurovascular coupling unit, which can predispose to ischemic circumstances resulting in structural brain abnormalities. This then can lead to dysfunction of neuronal networks, that, in turn, contributes to cognitive dysfunction and, ultimately, dementia (6, 7).

**Box 1 | The neurovascular coupling unit**

The neurovascular coupling unit is responsible for the regulation of the supply of sufficient levels of nutrients and oxygen to neuronal cells in the brain, in order to meet the local energy demand.

Intact function of the neurovascular coupling unit is crucial for optimal neuronal function because the brain lacks energy reserves and is dependent on a continuous and well-regulated delivery of oxygen and nutrients (such as glucose) through the cerebral blood supply (6, 7, 11–13).

**Figure 1:**
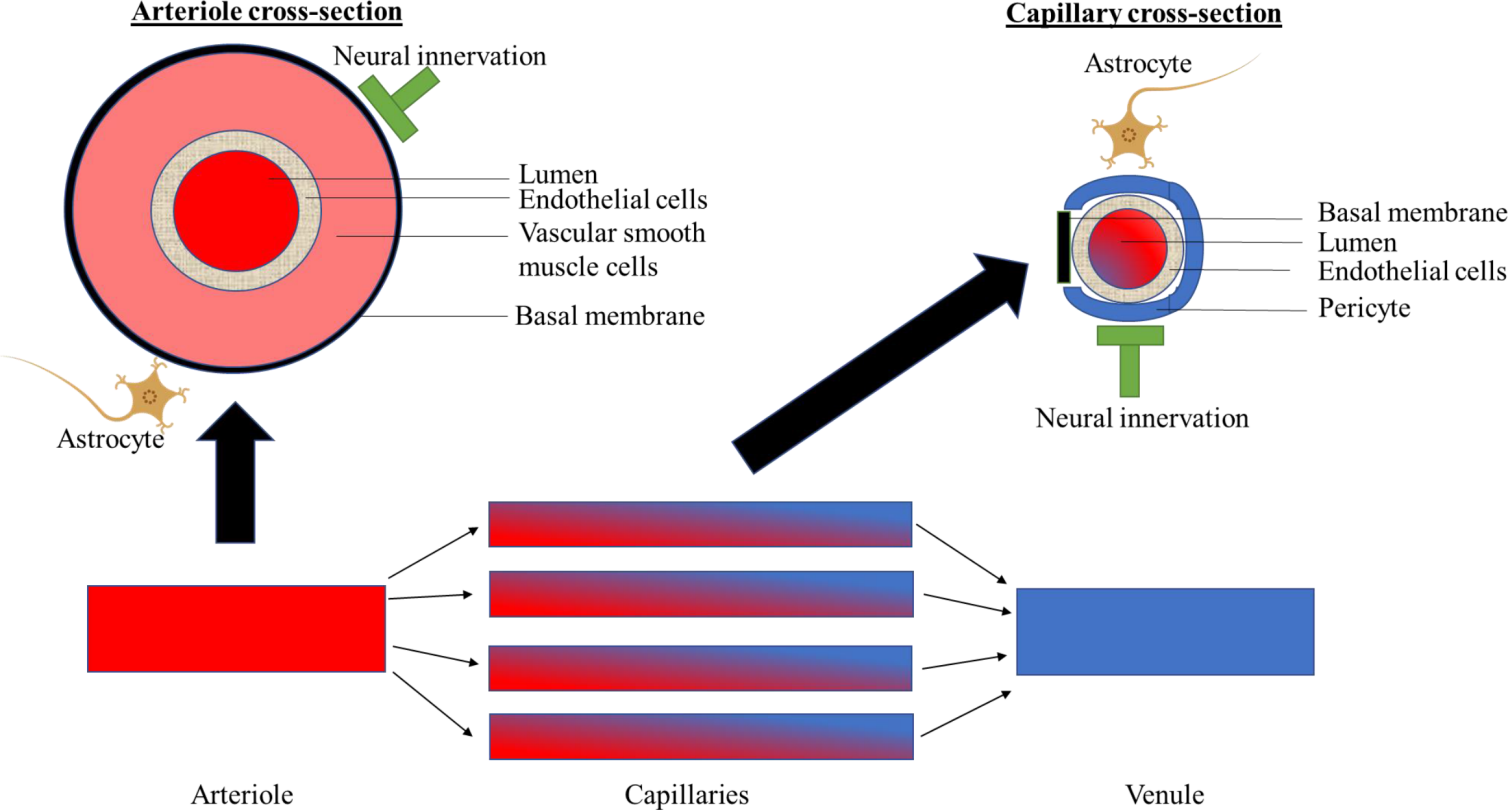
Schematically shows components of the neurovascular coupling unit at the level of the arterioles, capillaries and venules.

The neurovascular coupling unit comprises neuronal and microvascular structures that functionally interact both at the level of the arterioles as well as at the capillary level (blood-brain barrier) to regulate optimal metabolic conditions (**Figure 1**) (6, 7, 11–13). The neurovascular coupling unit, on the one hand, regulates the extent of blood flow to the capillaries (*via* regulation of the diameter of arterioles); and, on the other hand, regulates the integrity of the blood-brain barrier, which enables the maintenance of an optimal metabolic milieu for neurons (6, 7, 11–13). These functions are executed *via* bidirectional interactions between neuronal cells, i.e. neurons and glial cells (astrocytes, microglia, oligodendrocytes) and microvascular structures, i.e. endothelial cells, pericytes, and vascular smooth muscle cells (6, 7, 11–13). The exact communication among different cell types is reviewed elsewhere (6, 12). Briefly, neuronal cells can alter arteriolar diameter *via* transmitting signals to the endothelial cells in the capillaries, which *via* endothelial cell-to-cell connections can be transmitted to endothelial cells in the arterioles (i.e. neurovascular coupling). The arteriolar diameter is then modulated *via* nitric oxide (NO) release from endothelial cells to vascular smooth muscle cells (6, 7, 11–13). Microvascular structures, such as pericytes, may also contribute to the regulation of vascular smooth muscle cells *via* inducing NO release from neuronal cells (vasculo-neuronal coupling; e.g. *via* pericyte-astrocyte interactions) (6, 11, 12). Furthermore, the bidirectional interactions between neuronal and microvascular structures might also occur at the level of the venules (6, 7, 11–13).

At present, it remains incompletely understood how and to what extent dysfunction of the neurovascular coupling unit contributes to the early stage pathobiology of dementia (6, 7). Hence it is important to investigate: 1) how dysfunction of the neurovascular coupling unit is associated with cognitive performance; and 2) how degeneration of the components of the neurovascular coupling unit (i.e. neuronal and vascular structures) and loss of the interaction between neuronal and vascular structures are associated with cognitive performance.

To obtain such insight, it is important that large scale population-based evidence from humans is acquired (6, 7). Methodological strengths of population-based studies (in the present review defined as ~n>500) are that such studies provide data that is representative of (thus valid in) the general population; are less susceptible to selection bias than smaller studies which use clinical study populations; and are well-powered (i.e. due to the large size of the study population small associations can be detected) (8).

It is likely not feasible to acquire population-based data using currently existing cerebral imaging techniques. Disadvantages of cerebral imaging techniques are that these techniques are expensive, time-consuming, and do not allow the direct visualization of neurons and blood vessels (6, 7). In addition, a disadvantage of some, but not all, cerebral imaging techniques is that they are invasive (e.g. dynamic contrast-enhanced magnetic resonance imaging [DCE-MRI] requires the administration of exogenous contrast) (9).

As the retina has been postulated to be a window to the brain, imaging the retinal may provide opportunity to move towards quantitative population-based evidence on how and to what extent dysfunction of the neurovascular coupling unit contributes to the early pathobiology of cognitive dysfunction. The retina and brain share many anatomical and physiological similarities (10). Important advantages of retinal imaging are that retinal imaging techniques are non-invasive, highly accurate, relatively inexpensive, require relatively short measurement time, and can be used to directly visualize neuronal and microvascular components of the neurovascular coupling unit (10).

In this review, we demonstrate 1) that there is presently limited data on the association of dysfunction of neurovascular coupling unit, as estimated with cerebral imaging techniques, with cognitive performance; and 2) that there may be an opportunity to, using retinal imaging techniques, acquire population-based data on how and to what extent degeneration of components of the neurovascular coupling unit (i.e. neuronal and microvascular structures) and dysfunction of the neurovascular coupling unit are associated with early stage cognitive dysfunction. In section 1, we review the available human (population-based) evidence on the association of dysfunction of neurovascular coupling unit, as estimated with *cerebral* imaging techniques, with cognitive performance. In sections 2 and 3, we review the available human (population-based) evidence on the associations of neuronal and microvascular structures and function of the neurovascular coupling unit, as quantified by *retinal* imaging techniques, with cerebral magnetic resonance imaging (MRI) features of structural brain abnormalities (i.e. brain volume, white matter hyperintensities, lacunar infarcts, and cerebral microbleeds; section 2) and cognitive performance (section 3). A background on the neurovascular coupling unit is given in Panel 1 and **Figure 1**.

### Assessment of the function of the neurovascular coupling unit

Recently, a number of brain imaging techniques have been proposed to quantify neurovascular coupling unit function, as reviewed elsewhere (14). These include quantification of cerebral perfusion at rest and during neuronal activity, and quantification of blood-brain barrier function. Intact cerebral perfusion and blood-brain barrier function all presumably require intact function of the neurovascular coupling (14). Cerebral perfusion at rest can be assessed with single-photon emission computed tomography (SPECT) and arterial spin labelling (ASL)-MRI; cerebrovascular vasoreactivity can be assessed with blood oxygen level-dependent (BOLD) fMRI; and the integrity of the blood-brain barrier can be assessed with dynamic contrast enhanced (DCE)-MRI and the cerebrospinal fluid (CSF)/blood-albumin ratio (14).

### Pathophysiological mechanisms: Dysfunction of the neurovascular coupling unit and cognitive dysfunction

Impaired function of the neurovascular coupling unit may *via* several mechanisms lead to structural brain changes, which can lead to cognitive dysfunction (6, 7, 15). As postulated in the “Calcium Hypothesis”, cerebral blood flow deficits may lead to transient large increases of Calcium^2+^ (Ca^2+^) that can cause glutamate excitotoxicity and can lead to neurodegeneration (6, 7, 15). Additionally, cerebral blood flow deficits may impair the clearance of waste products from the neuronal tissue, which can result in the accumulation of toxic waste products in neuronal tissue, predisposing to degeneration of neuronal and microvascular structures (6, 7, 15). Further, deterioration of the integrity of the blood-brain barrier may result in the leakage of toxins from the circulation into neuronal tissue, such as amyloid beta, which may damage neuronal and microvascular structures (6, 7, 15).

### Causes of degeneration of components of the neurovascular coupling unit

Several potentially modifiable risk factors have been identified that may contribute to degeneration of neuronal and microvascular structures (16–19). Key factors are diabetes, midlife hypertension, midlife obesity, physical inactivity, and smoking, as reviewed elsewhere (16–19).

Of the neuronal components of the neurovascular coupling unit only neurons and astrocytes are shown. For a further explanation, please see Panel 1. Section 1: Summary of human evidence on the association of dysfunction of the neurovascular coupling unit, as quantified by cerebral imaging, with cognitive performance

In this section, we review the present body of evidence on the association of dysfunction of the neurovascular coupling unit, as quantified by lower cerebral perfusion at rest, lower cerebral vasoreactivity, and higher blood-brain barrier permeability, with cognitive performance. We searched for studies that estimated cerebral perfusion at rest from SPECT or ASL-MRI; cerebral vasoreactivity from BOLD fMRI; and blood-brain barrier dysfunction from DCE-MRI or CSF/blood-albumin ratio (for an explanation of these techniques, see Panel 1) (14). Details on the search strategy are given in the **Supplemental Methods**.

#### Literature overview

**Table 1** shows an overview of our findings. We did not identify any population-based studies (n>500) and, therefore, we show findings for smaller studies in **Table 1**. We identified three studies on cerebral perfusion at rest estimated with SPECT (one prospective study [n=103] and two cross-sectional studies [n=12-n=104]); four studies on cerebral vasoreactivity estimated with BOLD fMRI (one prospective study [n=116] and three cross-sectional studies [n=59-n=232]); 33 studies on cerebral perfusion at rest estimated from ASL-MRI (of these 33 studies we show the five largest studies [those with a study population sample size of n≥150] in **Table 1**; other studies are given in **Supplemental Table S2**]; of these five studies, one study was prospective [n=309] and four studies were cross-sectional [n=162-n=179]); nine studies on blood-brain barrier leakage estimated with DCE-MRI (two prospective studies [n=51 and n=57] and seven cross-sectional studies [n=45-n=102]); and three studies on blood-brain barrier leakage estimated with the CSF/blood albumin ratio (one prospective study [n=118] and two cross-sectional studies [n=45 and n=118]).

**Table 1:** Association of dysfunction of the neurovascular coupling unit, as quantified by cerebral imaging, with cognitive performance.

N	Study population	Brain imaging technique	Cognitive performance assessment	Confounders taken into account	Main results	Reference
Cerebral perfusion at rest or cerebrovascular vasoreactivity						
*Prospective studies*						
N=103 (2 years follow-up)	N= 35 patient with AD; n=33 patients with MCI; and n=35 elderly controls.	SPECT	Cognitive performance was assessed with the 15-objects test, which consists of the following: Temporal, Spatial and Personal Orientation; Digit span forwards and backwards, Block Design and Similarities subtests of Wechsler Adult Intelligence Scal (Third Edition); The Word List Learning test from the Wechsler Memory Scale (Third Edition); the RBANS visual memory subtest; Verbal comprehension (2 simple, 2 semi-complex and 2 complex commands); an abbreviated 15 item confrontation naming test from the Boston Naming Test; the Poppelreuter test; Luria’s Clock test; Ideomotor and Imitation praxis; the Automatic Inhibition subtest of the Syndrom Kurtz Test (SKT); Phonetic Verbal Fluency (words beginning with ‘P’ during one minute); Semantic Verbal Fluency (‘animals’ during one minute), and the Spanish version of the Clock Test.	None	The decline in performance on the 15-objects test between baseline and 2-year follow-up was significantly correlated (P= 0.001) with a lower cerebral perfusion at rest in the left Broadman Area 48.	Alegret, 2012 (20);
N=116 (4-year follow-up)	116 cognitively-normal adults ranging from 20 to 88 years old.	BOLD- fMRI	Four domains of cognitive function were assessed, i.e. processing speed, working memory, reasoning, and episodic memory. Processing speed was measured using the Digit Comparison Task, adapted from the Letter Comparison Task of Salthouse & Babcock, and Wechsler Adult Intelligence Scale-III Digit Symbol; working memory was evaluated from the Cambridge Neuropsychological Test Automated Battery Spatial Working Memory, and Wechsler Adult Intelligence Scale-III Letter Number Sequencing); reasoning was estimated using the Raven’s progressive matrices, ETS letter sets and Cambridge Neuropsychological Test Automated Battery Stockings of Cambridge; and episodic memory was assessed using the modified Hopkins Verbal Learning Task, and the Cambridge Neuropsychological Test Automated Battery Verbal Recognition Memory Task.	Baseline cerebrovascular vasoreactivity and baseline cognitive performance	Lower whole brain cerebrovascular vasoreactivity was significantly associated with a greater decline in processing speed (P<0.05) and episodic memory (P<0.05), but not with other domains.	Peng, 2018 (21);
N=309; 4-years follow-up	Individuals aged 20 to 89 years old.	ASL-MRI	Change in general cognitive performance over time was calculated, where general cognitive ability was estimated from four domains of cognitive function: processing speed, working memory, reasoning, and episodic memory. Processing speed was evaluated using the Digit Comparison Task, adapted from the Letter Comparison Task of Salthouse & Babcock, and Wechsler Adult Intelligence Scale-III Digit Symbol test. Working memory was measured using the Cambridge Neuropsychological Test Automated Battery Spatial Working Memory test and the Wechsler Adult Intelligence Scale-III Letter Number Sequencing test. Reasoning was estimated using Raven’s progressive matrices, ETS letter sets and the Cambridge Neuropsychological Test Automated Battery Stockings. Episodic memory was assessed using the modified Hopkins Verbal Learning Task and the Cambridge Neuropsychological Test Automated Battery Verbal Recognition Memory Task.	Age, systolic blood pressure, physical activity, and baseline global cognitive performance (or domain-specific performance in analyses with individual domains as outcome).	Greater whole-brain cerebral blood flow at baseline was significantly associated with greater general cognitive performance over time (P=0.031). Greater frontal cerebral blood flow at baseline was significantly associated with greater general cognitive performance over time in the older group (>53 years old; P=0.021). Cerebral blood flow in other brain lobes (occipital lobe, parietal lobe, and temporal lobe) was not associated with general cognitive performance. Additionally, in the older group (>53 years old) frontal cerebral blood flow was associated with reasoning ability (P=0.006) and episodic memory (P=0.009).	De Vis, 2018 (22);
*Cross-sectional studies*						
N=104	Men; 81y old; without dementia or stroke.	SPECT	Low versus high achievers, estimated from the MMSE, verbal ability, verbal memory, Digit Symbol Substitution Test, and the Benton Visual Retention Test.	None	Frontal, temporal, parietal, occipital, basal nucleus, thalamus, and subcortical regional cerebral blood flow were significantly lower in low achievers than high achievers.	André-Petersson, 2017 (23);
N=12	Patients >45 years old from a stroke center.	SPECT	Repeatable Battery for Assessment of Neuropsychological Status, which consisted of the following tests: the Digit Span subtest of the Wechsler Adult Intelligence Scale (third edition), Trail Making Test parts A and B, Clock Drawing Tests CLOX1 and CLOX2, and the Controlled Oral Word Association Test	None	Cerebral perfusion at rest in the left limbic lobe, right sub lobar lobe, right temporal lobe, left sub lobar lobe, right cerebellum (anterior lobe), and left frontal lobe were mostly all significantly correlated with the repeatable Battery for Assessment of Neuropsychological Status score.	Baker, 2013 (24);
N=78	n=24 individuals with MCI; and n=54 normal controls.	BOLD fMRI and ASL-MRI	MMSE	Age, sex, race, body-mass index, years of education, hypertension status, diabetic status, hypertension medication status, diabetic medication status, and either gray matter (for analyses in white matter regions) or white matter volume (for analyses in grey matter regions)	Cerebral blood flow was not associated with MMSE score (neither in the white matter nor in the gray matter). A higher MMSE score was significantly associated with a higher cerebrovascular vasoreactivity (in gray matter: beta per point higher MMSE score, 0.689 per unit change in end-tidal CO_2_, P = 0.005; and in white matter: beta per point higher MMSE score, 0.578 per unit change in end-tidal CO_2_, P= 0.016).	Kim, 2021 (25);
N =218	N=68 individuals with cognitive impairment and moderate to severe white matter hyperintensities; N=63 individuals without cognitive impairment and with no white matter hyperintensities; N=87 normal controls.	BOLD- fMRI	General cognitive performance was assessed with the MMSE, and a Chinese version of the MoCa. Executive function was assessed with the Trail Making Test-B and Stroop Color and Word Tests C (Stroop C); information processing speed was assessed with the Trail Making Test-A and Stroop Color and Word Tests A and B (Stroop A and B); memory was assessed with the Wechsler Memory Scale-visual reproduction-delayed recall and Auditory Verbal Learning Test-long delayed recall, representing visual memory and verbal memory, respectively; visuospatial performance was assessed with the clock drawing test and *via* a visual reproduction-copy test; and language abilities were assessed with the category verbal fluency test and Boston naming test.	Age, sex, years of education, and vascular risk factors (history of chronic diseases such as diabetes mellitus, hypertension, and dyslipidemia, and smoking status).	The group with cognitive impairment and white matter hyperintensities showed further decreased cerebrovascular vasoreactivity in the left frontal area, in comparison with the group with white matter hyperintensities without cerebrovascular vasoreactivity (P < 0.05). In the group with cognitive impairment and with white matter hyperintensities lower cerebrovascular vasoreactivity in the left frontal area was significantly associated with lower performance on tests for general cognition (r = 0.311), executive function (r = 0.362), and information processing speed (r = 0.399; all P < 0.05).	Ni,2020 (26);
N=59	N=30 younger healthy individuals (men and women; mean age 30 years); and n= 29 older healthy individuals (mean and women; mean age 65 years).	BOLD- fMRI	Cognitive performance (i.e. memory and attention) was assessed with the Swinburne University Computerized Cognitive Aging Battery.	Age, sex, and years of education	Lower cerebrovascular vasoreactivity in the temporal lobes was significantly associated with a lower memory score (P<0.05). In the older, but not the younger, group lower cerebrovascular vasoreactivity in the hippocampus was significantly associated with a lower memory score (P<0.05).	Catchlove, 2018 (27);
N=162	N=51 individuals with MCI; N=31 individuals with objectively-defined subtle cognitive decline; and N=80 individuals without cognitive impairment.	ASL-MRI	With MCI was defined as follows: >1 SD below the age-/education-/sex-adjusted mean on: (1) two neuropsychological measures within the same cognitive domain, or (2) at least one measure across all three sampled cognitive domains, or (3) a score of 6 or higher on the Functional Activities Questionnaire score. With objectively-defined subtle cognitive decline was defined as follows: 1 SD below the age-/education-/sex-adjusted mean on (1) one impaired total test score in two different cognitive domains (memory, language, attention/executive), or (2) two impaired neuropsychological process scores from the Auditory Verbal Learning Test, or (3) one impaired total test score and one impaired process score. Tests: Memory measures were the Rey Auditory Verbal Learning Test, delayed free recall correct responses and the Auditory Verbal Learning Test recognition test); language measures were: 30-item Boston Naming Test total correct and the Animal Fluency total score); and attention/executive functioning measures were: Trail Making Test Parts A and B.	None	Hippocampal cerebral blood flow was higher in individuals with objectively-defined subtle cognitive decline than in individuals without cognitive impairment (*P *= 0.007) or with MCI (P* *= 0.016). However, hippocampal cerebral blood flow did not differ between individuals with objectively-defined subtle cognitive decline and individuals with MCI (P* *= 0.974). A similar pattern was found for the inferior parietal lobe and the inferior temporal gyrus.	Thomas, 2020 (28);
N=232	N=33 individuals with AD; N= 87 individuals without dementia and who are amyloid positive; and N = 112 without dementia and who are amyloid negative.	ASL-MRI	Global cognitive performance was measured with the MMSE. Verbal memory encoding was assessed using the Rey Auditory Verbal Learning Test recognition subtest. Executive function was evaluated using Trails B of the Trail Making Test.	Age, APOe4 carrier status, BMI, sex, and fludeoxyglucose uptake	Cerebral blood flow was significantly lower in individuals with AD versus amyloid-negative individuals without dementia in the left hippocampus (*P* = 0.03) and left inferior temporal cortex (P = 0.01). In addition, cerebral blood flow was significantly lower in the inferior parietal cortex in individuals with AD than in individuals without AD who were amyloid positive (*P* < 0.001) or amyloid negative (*P* < 0.001). Associations of cerebral blood flow with MMSE score, verbal memory score and executive function score were not shown.	Yew, 2017 (29);
N=182	N=24 individuals with AD; N=66 individuals with early MCI; N=41 individuals with late MCI; and N=51 healthy controls.	ASL-MRI	MMSE	Age, sex and reference cerebral blood flow (precentral cortex cerebral blood flow).	Mini-Mental State Examination score was positively correlated with a higher cerebral blood flow in the entorhinal cortex (P *=* 0.034), the hippocampus (P *=* 0.028), and the inferior temporal (P *=* 0.0072) cortex. Cerebral blood flow was reduced in individuals with AD as compared with control subjects in most regions where effects were expected (entorhinal cortex, P *<* 0.01; hippocampus, P *=* 0.01; inferior temporal, P *<* 0.01; inferior parietal cortex, P *<* 0.01; posterior cingulate cortex, P *=* 0.07; precuneus, P *<* 0.01; medial-orbital frontal cortex, P *=* 0.03). There was no significant difference in cerebral blood flow between controls and individuals with early or late MCI.	Mattsson, 2014 (30);
N=179	N= 71 individuals with AD; N=35 patients with MCI; and N=73 subjects with subjective memory complaints who visited a memory clinic.	ASL-MRI	MMSE	Age, and sex	Both greater total and regional cerebral blood flow were significantly associated with a higher MMSE score (this association was mainly driven by the strong association between cerebral blood flow and cognitive performance within the AD group, most markedly in the parietal and precuneus and posterior cingulate regions). There were no significant differences in cerebral blood flow between individuals with MCI and individuals with subjective memory complaints (though values of cerebral blood flow were numerically in between those of individuals with subjective memory complaints and AD).	Binnewijzend, 2013 (31);
Blood-brain barrier						
*Prospective studies*						
N=51; 2-years follow up.	Patients with lacunar stroke or MCI.	DCE-MRI	Global cognitive performance was estimated from memory, executive function, and information processing speed.	Age, sex, educational level, baseline white matter hyperintensity volume, and baseline brain volume	Greater blood-brain barrier leakage rate was significantly associated with a greater cognitive decline (in normal white matter, standardized beta [95% CI]: 0.65 [0.065–1.23]; and in grey matter: 1.34 [0.64–2.00]).	Kerkhofs, 2021 (32);
N=57 12-years follow-up.	Community-dwelling participants	DCE-MRI	Cognitive performance was estimated from memory function, processing speed, executive functioning, and executive functioning. Memory function was measured using the verbal learning test in which the immediate recall score gives an indication of short-term episodic memory and learning, and the delayed recall score is considered to be a measure of long-term episodic memory. Processing speed was measured with the letter-digit substitution test. Executive functioning was measured with the Stroop color-word test, in which the interference score is considered to be a measure of inhibition.Cognitive decline was calculated by subtracting the participants’ current score from their previous score in the last measure approximately 12 years ago, so that a larger difference score would correspond to more cognitive decline.	Age, sex, and education level	There was a significant association between blood-brain barrier leakage (k_i_) in the white and grey matter and decline in delayed recall (white matter, beta per 10^−6^ · min^−1^ greater leakage 0.389 SD, P = 0.006; grey matter, beta per 10^−6^ · min^−1^ greater leakage = 0.287, P = 0.044).	Verheggen,2020 (33);
N=118; 34-months follow up	Community-dwelling individuals	CSF/blood albumin	Cognitive decline was assessed with the Clinical Dementia Rating sum of boxes.	Age, sex, APOe4 status, and baseline clinical dementia rating	Each unit increase in the CSF/blood albumin index was significantly associated with an increase in the Clinical Dementia Rating–Sum of Boxes by 0.09 units (P = .015).	Bowman, 2018 (34);
*Cross-sectional*						
N=80	N=14 patients with AD; N=34 patients with MCI; and N=32 normal controls.	DCE-MRI	Memory and information processing speed.	Age, sex, educational level, lag time between MRI and neuropsychological assessment, diagnostic group	Greater blood-brain barrier leakage rate was significantly associated with lower information processing speed (P<0.05), and in the same direction, but not statistically significantly, associated with lower memory function.	Freeze, 2020 (35);
N=47	N=26 patients with MCI; and N=21 normal controls.	DCE-MRI	Overall cognitive performance assessed with the MoCA.	Age, sex, vascular risk factors, education	Greater leakage rate (per 10^−4^ min^−1^) was significantly associated with lower MoCA score (in points) in white matter hyperintensities (beta, −4.363 [95% CI NR], but not significantly associated with lower MoCa score (in points) in normal white matter volume (-4.718 [95%CI NR]); cortical grey matter (-0.571 [95%CI NR]); and in deep grey matter (-1.346 [95%CI NR]).	Li, 2021 (36);
N=75	N=39 patients with MCI; and n=36 normal controls.	DCE-MRI	MMSE, Clinical Dementia Rating Scale global and Sum of Boxes Score.	Education level	In the female MCI group greater blood-brain barrier leakage (K_trans_) of the occipital cortex was significantly associated with lower MMSE score (beta, per 10^−3^ min^−1^,−0.397 points, P = 0.025). However, there was not association with the Clinical Dementia Rating Scale global and Sum of Boxes Score. The difference in blood-brain barrier leakage between individuals with MCI and healthy controls was not reported.	Moon, 2021 (37);
N=102	N=36 with low white matter hyperintensity burden; N=35 with medium white matter hyperintensity burden; and N=31 with a high white matter hyperintensity burden. Individuals were enrolled from a neurology clinic.	DCE-MRI	MMSE, MoCA.	Age, sex, education years, and white matter hyperintensity burden	Scores on MMSE and MoCA decreased with increasing leakage rate in white matter hyperintensities (beta per unit increase in K_i_ −0.857 points, P = 0.029; and −1.492, P = 0.006; respectively) and in deep grey matter (beta, −1.216, P = 0.024; and −1.875, P = 0.012).	Li, 2017 (38);
N=45	N=21 patients with MCI; and N=24 normal controls.	DCE-MRI	Diagnostic criteria were not reported	None	In individuals with MCI, as compared to age-matched older normal controls, there was a significant increase in the blood-brain barrier permeability (K_trans_) in the hippocampus and its CA1 and dentate gyrus regions, but not CA3. In individuals with MCI, as compared to age-matched older normal controls, there were no significant differences in the blood-brain barrier permeability in cortical, subcortical and white matter regions.	Montagne, 2015 (39);
N=70	N=53 patients with vascular cognitive impairment; and N=17 normal controls.	DCE-MRI	Classified as with or without vascular cognitive impairment.	None	In individuals with vascular cognitive impairment DCE-MRI assessed permeability was significantly greater than in healthy controls (P<0.05).	Taheri, 2011 (40);
N=45	N=21 patients with MCI; and N=24 normal controls.	CSF/blood albumin	Diagnostic criteria not reported.	None	In individuals with MCI, as compared to age-matched older normal controls, there was a significant 30% increase in the CSF/blood albumin ratio.	Montagne, 2015 (39);
N=70	N=53 patients with vascular cognitive impairment; and N=17 normal controls.	CSF/blood albumin	Classified as with or without vascular cognitive impairment.	None	The CSF/blood albumin index was significantly higher in the individuals with vascular cognitive impairment than in the healthy controls (P<0.05)	Taheri, 2011 (40);
N=118	Community-dwelling individuals	CSF/blood albumin	Cognitive state was classified using the Clinical Dementia Rating	None	The CSF/blood albumin index was higher in individuals with MCI versus individuals without cognitive impairment (P = 0.005).	Bowman, 2018 (34);

**Table 1** shows the association of dysfunction of the neurovascular coupling unit, as quantified by cerebral imaging, with cognitive performance.

Fully adjusted results are shown (where appropriate, i.e. when associations where adjusted for confounders).

AD, Alzheimer’s disease; MCI, mild cognitive impairment; MRI, magnetic resonance imaging; MMSE, mini-mental state examination; MoCA, Montreal Cognitive Assessment; BOLD, blood oxygen level dependent; ASL, arterial spin labelling; DCE, dynamic contrast enhanced; CSF, cerebrospinal fluid; BMI, body-mass index; Apoe4, apolipoprotein E4; NR, not reported; SD, standard deviation; CI, confidence interval.

#### Summary of findings

Most studies found that lower cerebral perfusion at rest and lower cerebrovascular vasoreactivity (as quantified with SPECT, BOLD fMRI, and/or ASL-MRI) and higher blood-brain barrier leakage (as quantified with DCE-MRI or CSF/blood albumin ratio) were associated with lower cognitive performance. These findings support the concept that dysfunction of the neurovascular coupling unit may contribute to the early pathobiology of cognitive dysfunction.

#### Limitations of the evidence

The current evidence has certain methodological limitations. First, most studies were relatively small and included clinical samples only. Data from such smaller studies is relatively less representative of the general population than large population-based studies, thus the generalizability of findings to the general population is limited (8). Second, most studies were cross-sectional, hence these studies do not allow to account for temporality, which is an important criterion for the inference of causality (8). Third, most studies did not adjust in the analysis for potentially important confounders, including cardiovascular risk factors such as blood pressure or hyperglycemia; and lifestyle factors such as physical activity or diet. This may have led to an overestimation of the strength of the association between dysfunction of the neurovascular coupling unit and cognitive performance (8).

Section 2: Summary of human evidence on the associations of neuronal and microvascular structures and function of the neurovascular coupling unit, as quantified by retinal imaging, with cerebral MRI features of structural brain abnormalities

**Box 2** gives a background on biological similarities between the retina and brain; and Panel 2 and **Figure 2** provide an overview of retinal imaging techniques for the assessment of neuronal and microvascular structures and the function of the neurovascular coupling unit.

**Box 2 | Biological similarities between the retina and brain**

The retina and the brain have a shared embryology, and have a similar physiology, anatomy, and pathophysiology (10). Embryologically, both the retina and the brain are derived from the neural ectoderm; the retina invaginates from the diencephalon around 23 days of gestation (10). Next, the neurovascular coupling unit in the retina and the brain have similar functions. Like in the brain, neurovascular coupling at the level of the retina regulates cerebral perfusion at rest and during neuronal activity, and it maintains the integrity of the blood-retina barrier, a barrier which is analogous to the blood-brain barrier (10). Further, the same cell types control function of the neurovascular unit in the retina and the brain; and these cells use the same biological mechanisms to control these processes, as reviewed elsewhere (10). For example, both retinal and cerebral neurons use the neurotransmitters glutamate, gaba-aminobutyric acid (GABA), acetylcholine, dopamine, and glycine for intrasynaptic signaling; retinal and cerebral capillaries have similar tight junctions; capillary branching angles; and pericyte-to-endothelial cell ratio (10). Last, the pathophysiological mechanisms that underlie degeneration of components of the neurovascular coupling unit in the brain and retina have been found to be similar (10). For example, amyloid beta accumulation, a hallmark feature of Alzheimer’s disease, has been found in the retina as well as in the brain of individuals with Alzheimer’s disease (10).

In this section, we review the present body of evidence on the associations of neuronal and microvascular structures and function of the neurovascular coupling unit, as quantified by retinal imaging, with cerebral MRI features of structural brain abnormalities. We searched for studies that assessed features of cerebral small vessel disease (i.e. brain volume; white matter hyperintensities, cerebral microbleeds, and lacunar infarcts) (14) and retinal neurodegeneration (assessed as lower retinal nerve fiber layer [RNFL] thickness); retinal microvascular dysfunction (assessed as narrower or wider retinal arterioles and wider retinal venules); or retinal dysfunction of the neurovascular coupling unit (assessed as lower flicker light-induced increase in retinal microvascular diameters; lower perfusion of arterioles and venules at rest [assessed as lower velocity]; or lower capillary perfusion at rest [assessed as greater foveal avascular zone area or lower vascular density]) (10, 17). Details on the search strategy are given in the **Supplemental Methods**.

### Retinal imaging techniques for the assessment of neuronal and microvascular structures and the function of the neurovascular coupling unit

We discuss the following retinal imaging tools as a means for the quantification of neuronal and microvascular structures, respectively: optical coherence tomography (OCT) and fundoscopy; and the following retinal imaging tools as a means for the quantification of the function of the neurovascular coupling unit in the retina: dynamic vessel analyzer, retinal laser Doppler flowmetry, and OCT angiography (OCT-A) (10).

#### Optical coherence tomography

**Figure 2.1:**
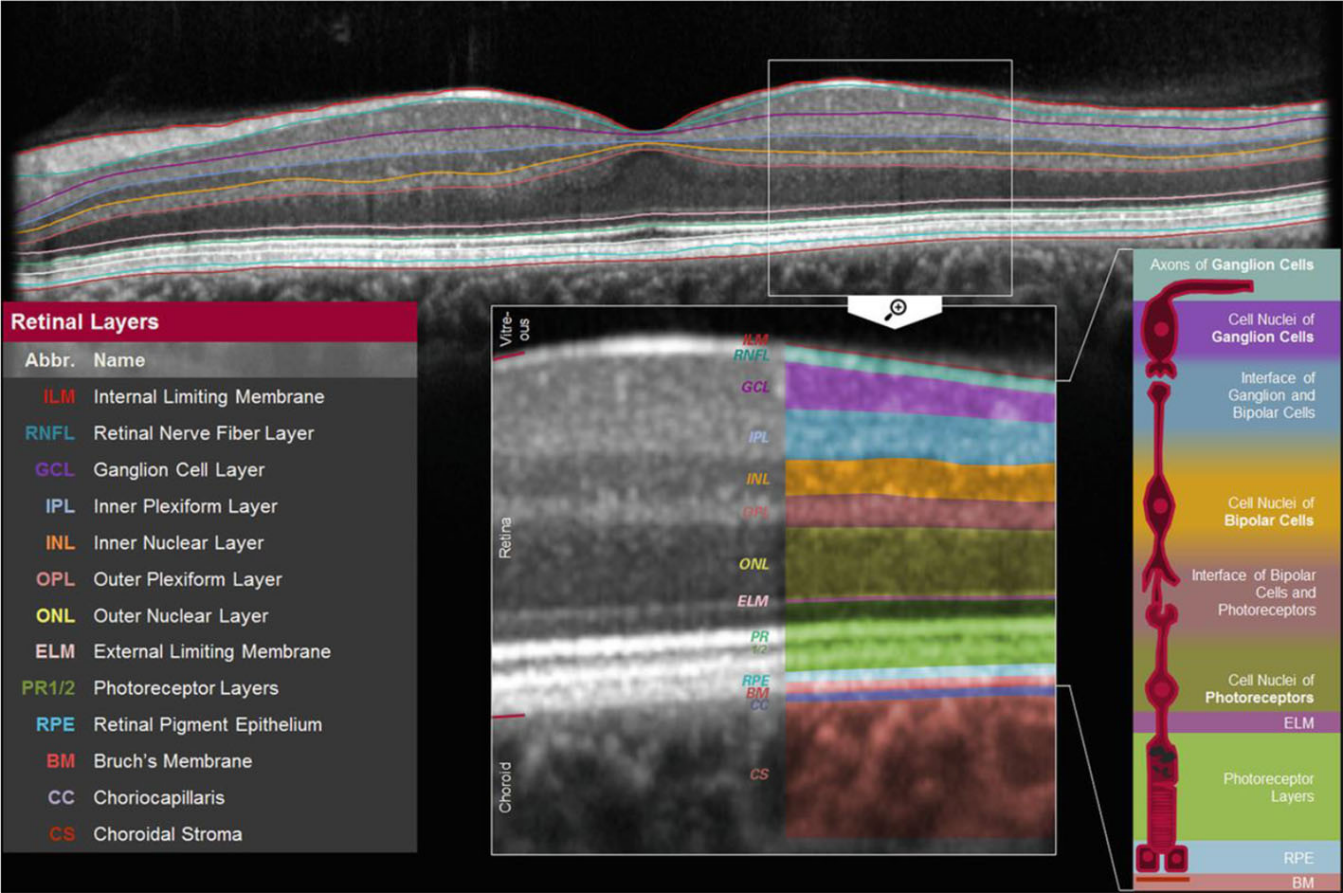
Macular (perifoveal) RNFL thickness. **Figure 2.1** shows a cross-section of the macula which shows the thickness of individual retinal layers, including the thickness of the retinal nerve fiber layer [adapted from (41)].

**Figure 2.2:**
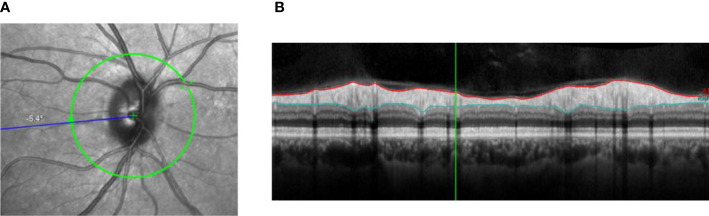
Peripapillary RNFL thickness. **(A)** Shows the location where the thickness of the RNFL can be determined around the optic nerve. Figure 2.2B shows the contours of the RNFL (red and light blue lines). **(B)** Shows the contours of the RNFL (red and light blue lines).

The thickness of individual retinal layers, including the RNFL, can be assessed with OCT (10). RNFL thickness is thought to reflect the number of retinal ganglion cell axons (thus a lower RNFL thickness indicates more neurodegeneration) and can be assessed in the central macular area (“perifoveal”; **Figure 2.1**) as well as around the optic nerve head (“peripapillary”; **Figure 2.2**) (10).

#### Fundoscopy

**Figure 2.3:**
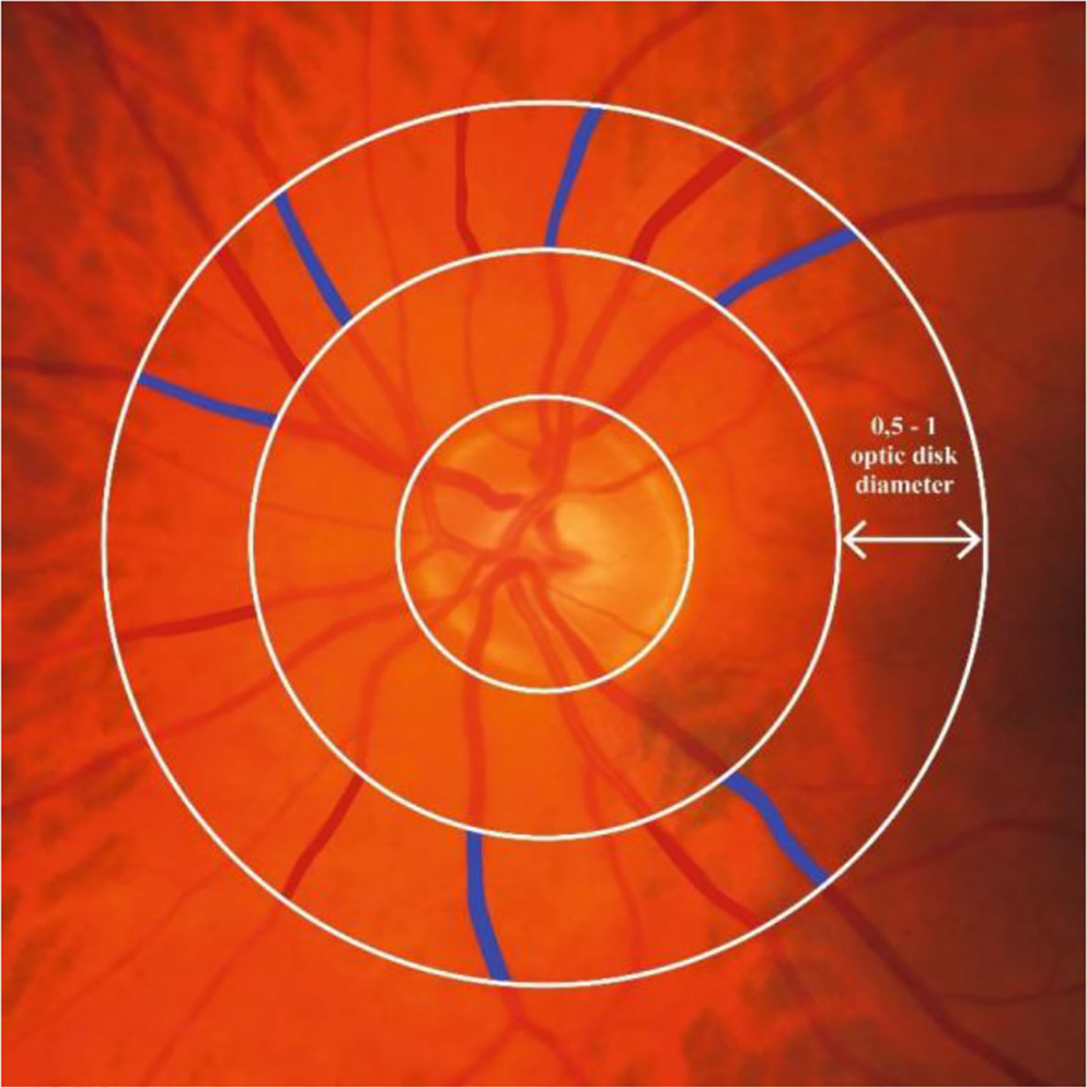
Retinal microvascular diameters. **Figure 2.3** shows where retinal arteriolar and venular diameters can be assessed around the optic nerve. Red and blue color indicate the area on the fundus photo covered by an arteriole and venule, respectively, at 1 disk diameter from the optic nerve head. Retinal arteriolar and venular diameters are calculated as the average diameter of six arterioles or six venules, respectively.

The diameter of retinal arteriolar and venular blood vessels around the optic nerve head can be assessed from fundus images (**Figure 2.3**) (10). Narrower or wider retinal arterioles and wider retinal venules are thought to reflect more microvascular dysfunction (10, 17).

#### Dynamic vessel analyzer

**Figure 2.4:**
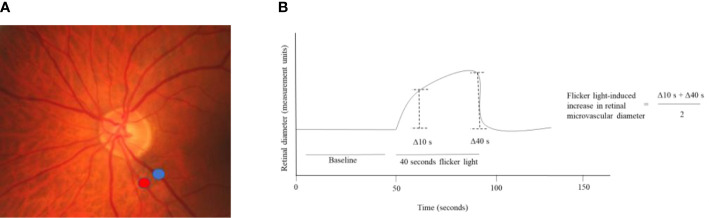
Flicker light-induced increase in retinal microvascular diameters. **(A)** Shows where the increase in retinal arteriolar (red) and venular (blue) diameters in response to flicker light exposure can be assessed around the optic nerve head. **(B)** Shows that the baseline diameter is assessed during the first 50 seconds of the measurement and that during 40 seconds of flicker light the retinal diameter increases. The flicker light-induced increase in retinal diameters is calculated from the retinal diameter at 10 and 40 seconds.

The dynamic vessel analyzer is a device that can quantify the change in retinal peripapillary arteriolar and venular diameters in response to exposure to flicker light (**Figure 2.4**) (42, 43). Flicker light is presumed to increase the metabolic demand of the retina, inducing an increase in retinal perfusion, which leads to widening of retinal arteriolar and venular diameters (42, 43). As the increase in retinal microvascular diameter is presumed to rely on the function of the neurovascular coupling unit, a greater increase in retinal arteriolar and venular diameter in response to flicker-light exposure is presumed to reflect better function of the neurovascular coupling unit (42, 43).

#### Retinal laser Doppler flowmetry

**Figure 2.5:**
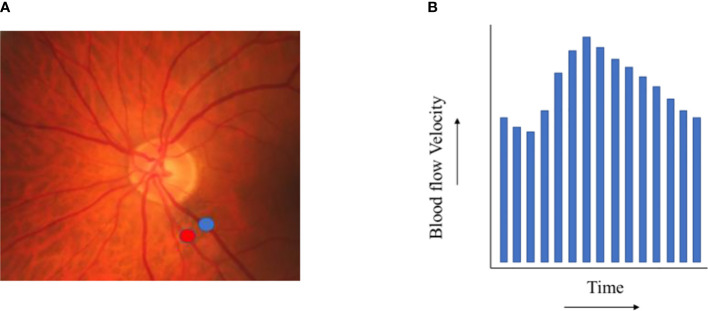
Retinal blood flow. **(A)** shows exemplary locations on a fundus photo where retinal arteriolar (red) and venular (blue) blood flow velocity can be assessed. **(B)** depicts the results of a retinal blood flow velocity measurement (during one heart beat).

Retinal laser Doppler flowmetry can be used to quantify the velocity of retinal blood flow in the arterioles and venules (**Figure 2.5**) (43). Lower retinal arteriolar and venular perfusion at rest is presumed to reflect worse function of the neurovascular coupling unit (43).

#### OCT-A

**Figure 2.6:**
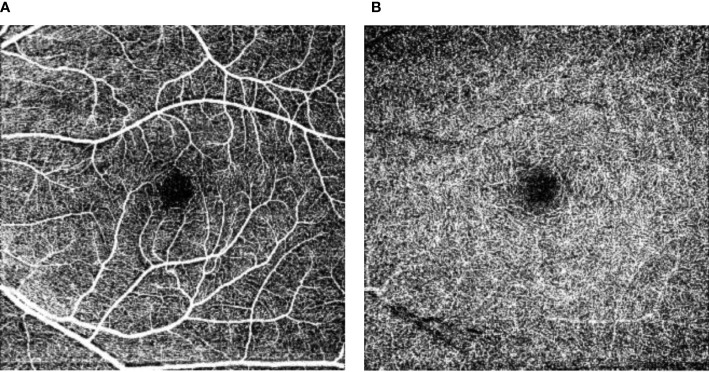
Retinal vascular density and foveal avascular zone. **Figure 2.6** shows the superficial capillary networks **(A)** and the deep capillary networks **(B)** in the perifoveal area. The central black area is the perifoveal avascular zone, where a larger perifoveal avascular area reflects less capillary perfusion at rest. In addition, from these images the capillary perfusion at rest can be estimated as the “vascular density”.

OCT-A can be used to visualize the retinal capillaries (10) and can quantify capillary perfusion at rest based on the size of the foveal avascular zone area and vascular density (**Figure 2.6**). A greater foveal avascular zone area and a lower vascular density are thought to both reflect lower capillary perfusion at rest (10).

#### Literature overview

**Table 2** shows the results of our search. We identified two population-based studies that investigated the associations of RNFL thickness with cerebral MRI features of structural brain abnormalities (both cross-sectional studies [n=2,124 and n=2,131]); and five population-based studies that investigated the associations of retinal arteriolar and venular diameters with cerebral MRI features of structural brain abnormalities (all cross-sectional studies[n=1,013-n=1,211]; an overview of smaller studies is given in **Supplemental Table S3**). We did not identify any studies (neither population studies nor smaller clinical studies) that investigated the associations of flicker light-induced increase in retinal microvascular diameters or retinal arteriolar or venular perfusion at rest with cerebral MRI features of structural brain abnormalities. We identified five smaller clinical studies that investigated the associations of capillary perfusion at rest, estimated as vascular density or foveal avascular zone area, with cerebral MRI features of structural brain abnormalities (all cross-sectional studies; n=16-n=85; as there were no population-based data available we showed findings of these smaller studies in **Table 2**).

**Table 2:** Associations of neuronal and microvascular structures and function of the neurovascular coupling unit, as quantified by retinal imaging, with cerebral MRI features of structural brain abnormalities.

N	MRI features of structural brain abnormalities	Confounders	Main results	Reference
RNFL thickness				
*Prospective studies*				
No studies available				
*Cross-sectional studies*				
N=2,124	Grey and white matter brain volume, cerebral microbleeds (n=352), lacunar infarcts (n=145).	Age, sex, sub cohort, education, axial length of the eye, intracranial volume, mean arterial blood pressure, blood pressure lowering medication, body mass index, total cholesterol, diabetes mellitus, and smoking	Lower peripapillary RNFL thickness was significantly associated with lower gray and white matter volume (beta per SD decrease in peripapillary RNFL thickness for the standardized mean difference in grey and white volume [95% CI], respectively, -0.03 [-0.05; -0.01] and -0.03 [-0.06; -0.01]). However lower RNFL thickness was not associated with the presence of cerebral microbleeds or lacunar infarcts (per SD lower RNFL thickness, OR [95%CI] 1.02 [0.90–1.15] and 1.11 [0.94–1.31], respectively).	Mutlu, 2017 (44);
N=2,131	Total brain volume	Age, age squared and sex, race, education, body mass index, mean arterial blood pressure and smoking	Lower macular RNFL thickness was non-significantly associated with lower total brain volume (beta, per SD decrease in macular RNFL thickness, standardized mean difference [95% CI], -0.03 [-0.07; 0.00]).	Chua, 2021 (45);
Retinal arteriolar diameter				
*Prospective studies*				
No studies available				
*Cross-sectional studies*				
N=1,211	Presence of lacunar infarcts (=200); Presence of white matter lesions (n= 384).	Age, sex, study site, hypertension, hypercholesterolemia, diabetes status and cigarette-smoking status.	Individuals with arteriolar narrowing (no definition provided), versus individuals without arteriolar narrowing, had a higher odds of the presence of a lacunar infarct (odds ratio [95% CI] for with versus without narrowing, 1.61 [1.01-2.57], but did not have a higher odds of the presence of white matter lesions (odds ratio [95% CI] for with versus without narrowing 1.24 [0.82; 1.87]) or for the presence of both a lacunar infarct and white matter lesions (odds ratio [95% CI] for with versus without narrowing 1.40 [0.86; 2.28]).	Liew, 2012 (46);
N=1,185	Presence of white matter hyperintensities (2% had none; 36% had one white matter hyperintensity; 31% had two white matter hyperintensities; 31% had three or more white matter hyperintensities).	Age, sex ethnicity, hypertension, body-mass index, glycated hemoglobin, diabetes, total cholesterol, high density lipoprotein cholesterol, log C-reactive protein, coronary artery disease, years of education and smoking habit, presence of retinopathy	Narrower arteriolar diameter was significantly associated with a lower odds of the presence of white matter hyperintensities (odds ratio [95% CI] per micrometer lower retinal arteriolar diameter 0.98 [0.97; 0.99]).	Hughes, 2016 (47);
N=1,065	Gray and white matter volume.	Age, sex, cohort, and the other vessels, venular diameter, systolic blood pressure, diastolic blood pressure, body-mass index, total cholesterol, high-density lipoprotein cholesterol, diabetes mellitus, and smoking	Wider retinal arteriolar diameter was not associated with gray and white matter volume (beta per SD increase in retinal arteriolar diameter for the standardized mean difference in grey and white volume [95% CI], respectively, -0.02 [-0.09; 0.06] and 0.04 [-0.03; 0.11]).	Ikram, 2013 (48);
N=1,013 (acute stroke patients)	Cortical and subcortical cerebral atrophy (n=424).	Age, sex, study site, hypertension, hypercholesterolemia, diabetes, and smoking status	Narrow retinal arteriolar diameter (defined as lowest quintile versus other quintiles combined as reference group) was not significantly associated with cortical and subcortical cerebral atrophy (narrow versus wide retinal arteriolar diameter, OR [95%CI] 1.3 [0.9-2.0]).	Baker, 2010 (49);
N=1,114 (N=557 stroke cases and N=557 controls)	Lacunar stroke (n=261).	Age, sex, race, smoking status, hypertension status, diabetes mellitus status, and hypercholesterolemia status	Narrower retinal arteriolar diameter (per SD) was significantly associated with a greater odds for the presence of lacunar stroke (per SD narrower retinal arteriolar diameter OR [95%CI] 2.87 [2.19-3.76]).	Ong, 2013 (50);
Retinal venular diameter				
*Prospective studies*				
No studies available				
*Cross-sectional studies*				
N=1,211	Presence of lacunar infarcts (=200); Presence of white matter lesions (n= 384).	Age, sex, study site, hypertension, hypercholesterolemia, diabetes status and cigarette-smoking status	Individuals with venular widening (no definition provided), versus individuals without venular widening, had a higher odds for both the presence of a lacunar infarct and white matter lesions (odds ratio [95% CI 2.27 [1.41; 3.654], though these associations were not statistically significant for either the presence of a lacunar infarct (odds ratio 1.39 [0.87-2.20], or the presence of white matter lesions (odds ratio [95%CI] 1.44 [0.95; 2.17]).	Liew, 2012 (46);
N=1,185	Presence of white matter hyperintensities (2% had none; 36% had one white matter hyperintensity; 31% had two white matter hyperintensities; 31% had three or more white matter hyperintensities).	Age, sex ethnicity, hypertension, body-mass index, glycated hemoglobin, diabetes, total cholesterol, high density lipoprotein cholesterol, log C-reactive protein, coronary artery disease, years of education and smoking habit, presence of retinopathy	Wider venular diameter (per micrometer) was not associated with the odds of the presence of white matter hyperintensities (odds ratio [95% CI] 1.00 [0.99; 1.01]).	Hughes, 2016 (47);
N=1,065	Gray and white matter volume	Age, sex, cohort, and the other vessels, arteriolar diameter, systolic blood pressure, diastolic blood pressure, body-mass index, total cholesterol, high-density lipoprotein cholesterol, diabetes mellitus, and smoking	Wider retinal venular diameter was borderline significantly associated with white matter volume, but not with gray matter volume (beta per SD increase in retinal venular diameter for the standardized mean difference in white and grey volume [95% CI], respectively, -0.07 [-0.14; 0.00] and 0.06 [-0.02; 0.13]).	Ikram, 2013 (48);
N=1,013 (acute stroke patients)	Cortical and subcortical cerebral atrophy (n=424).	Age, sex, study site, hypertension, hypercholesterolemia, diabetes, and smoking status	Wide retinal venular diameter (defined as highest quintile versus other quintiles combined as reference group) was not significantly associated with cortical and subcortical cerebral atrophy (wide versus narrow retinal venular diameter, OR [95%CI] 1.3 [0.8-2.0]).	Baker, 2010 (49);
N=1,114 (N=557 stroke cases and N=557 controls)	Lacunar stroke (n=261).	Age, sex, race, smoking status, hypertension status, diabetes mellitus status, and hypercholesterolemia status	Wider retinal venular diameter (per SD) was significantly associated with a greater odds for the presence of lacunar stroke (per SD wider retinal venular diameter OR [95%CI] 1.60 [1.25-2.06]).	Ong, 2013 (50);
Arteriole-to-venule ratio				
*Prospective studies*				
No studies				
*Cross-sectional studies*				
N=1,635	Cerebral infarct (defined as a lesion ≥3 mm in maximum diameter in a vascular distribution with typical imaging characteristics).	Age, race, center, sex, 6-year mean arterial blood pressure, antihypertensive medication use, fasting blood glucose, diabetes, smoking status, plasma total and high-density lipoprotein cholesterol, plasma triglycerides, and serum fibrinogen	In individuals with hypertension a low arteriole-to-venule ratio (defined as lowest quintile), versus high arteriole-to-venule ratio (defined as highest quantile) was significantly associated with a higher odds of a cerebral infarct (odds ratio [95%CI] 3.74 [1.51; 9.24]; however this association was not significant in individuals without hypertension (odds ratio [95%CI] 0.64 [0.22; 1.87]).	Cooper, 2006 (51);
Flicker light-induced increase in retinal microvascular diameters				
*Prospective studies*				
No studies				
*Cross-sectional studies*				
Retinal blood flow				
*Prospective studies*				
No studies				
*Cross-sectional studies*				
OCT-A				
*Prospective studies*				
No studies				
*Cross-sectional studies*				
N=85; (N=46 individuals with a recent subcortical infarction; and N=39 healthy controls).	A cerebral small vessel disease composite score was calculated (based on the presence of lacunes, white matter hyperintensities, cerebral microbleeds, and enlarged perivascular spaces).	Age, sex, systolic and diastolic blood pressure, diabetes, dyslipidemia	Individuals with a recent subcortical infarction had a significantly lower length of micro vessels of the perfused inner retina per unit area in millimeters squared (mm^2^) than healthy controls (mean difference [95%CI] between groups was −0.087 [−0.145; -0.029] mm^2^). Greater inner retina length of micro vessels of the perfused inner retina per unit area in millimeters squared (mm^2^) was significantly associated with greater cerebral small vessel disease burden (beta [95%CI] per point for the cerebral small vessel disease burden score, 0.067 [0.013–0.121]).	Kwapong. 2022 (52);
N=77; (N=47 individuals with cerebral small vessel disease features; and N=39 healthy controls).	Cerebral small vessel disease was assessed based on the presence of lacunar infarcts, and white matter hyperintensities (white matter hyperintensities were assessed with the Fazekas score).	Age, hypertension, diabetes, and hyperlipidemia	Greater temporal superficial retinal capillary plexus vascular density was significantly associated with a lower periventricular white matter hyperintensities Fazekas score and a lower deep white matter hyperintensities Fazekas score (beta per mm greater vascular density, -1.772 [-3.048; -0.497]; and -1.427 [-2.679; -0.175]); however, there was no significant association with less lacunar infarcts -0.160 [-0.373; 0.054]). However, these associations were not found when the radial peripapillary capillary network was investigated.	Wang, 2021 (53);
N=74; all individuals without stroke or dementia.	White matter hyperintensities were scored according to the Fazekas score.	Age, sex, hypertension, and signal strength index	Participants with Fazekas score ≥2, versus participants with Fazekas score ≤ 1, had a significantly lower radial peripapillary capillary network density (*P* = 0.02) and deep retinal capillary plexus network density (*P* = 0.012), but did not have a significantly lower superficial retinal capillary plexus network.	Peng, 2020 (54);
N=69; (N=29 individuals with amyloid-positive AD-related cognitive impairment; N=25 individuals with subcortical vascular cognitive impairment; and N=15 amyloid-negative cognitively normal individuals).	The following cerebral small vessel disease features were assessed: presence of lacunes, white matter hyperintensities, and cerebral microbleeds.	Age, sex, hypertension, diabetes, and image quality score	In individuals with subcortical vascular cognitive impairment, versus normal controls, the capillary density in the inferior radial peripapillary capillary network was significantly lower (P=0.001). However, individuals with amyloid-positive AD-related cognitive impairment, versus normal controls, the capillary density in the inferior radial peripapillary capillary network was not significantly lower (P>0.05).	Lee, 2020 (55);
N=16; (N=7 individuals with MCI; and N=9 individuals with AD).	Forebrain parenchyma, cortical gray matter, inferolateral ventricle, lateral ventricle, and hippocampus volumes.	None	Inferolateral ventricle volume inversely correlated with the vascular density in the 6-mm circle (ρ = −0.565, *P* = .028) and 3-mm ring (ρ = −0.569, *P* = .027) and the perfusion density in the 3-mm ring (ρ = −0.605, *P* = .0169). Forebrain, cortical gray matter, later ventricle, and hippocampus volumes did not significantly correlate with either vascular density or perfusion density (*P* >.05).	Yoon, 2019 (56);

**Table 2** shows the associations of neuronal and microvascular structures and function of the neurovascular coupling unit, as quantified by retinal imaging, with cerebral MRI features of structural brain abnormalities.

The following MRI features were considered: total brain volume, white matter hyperintensities, lacunar infarcts, and cerebral microbleeds. Fully adjusted results are shown (where appropriate, i.e. when associations where adjusted for confounders).

RNFL, retinal nerve fiber layer thickness; NR, not reported; SD, standard deviation; CI, confidence interval; MCI, mild cognitive impairment; AD, Alzheimer’s disease.

#### Summary of findings

Most population-based studies found that lower RNFL thickness, narrower retinal arteriolar diameters, and wider retinal venular diameters were associated with a higher prevalence of cerebral MRI features of structural brain abnormalities. In addition, most studies (all small and non-population-based) found that lower capillary perfusion at rest, as estimated from lower vascular density, was associated with a higher prevalence of cerebral MRI features of structural brain abnormalities. Next, no studies have been reported that investigated the associations of flicker light-induced increase in retinal microvascular diameters, retinal arteriolar or venular perfusion at rest, or capillary perfusion at rest, as estimated based on the size of the foveal avascular area, with cerebral MRI features of structural brain abnormalities. Overall, these findings are consistent with the concept that the retinal imaging techniques may provide an opportunity to study the early pathobiology of cognitive dysfunction.

#### Limitations

The present body of evidence has certain limitations. The main limitation of the available population-based data on RNFL thickness and retinal microvascular diameters is that most studies were cross-sectional in design (8). In addition, data were lacking for flicker light-induced increase in retinal microvascular diameters, retinal blood flow at rest, and capillary perfusion estimated from the size of the foveal avascular zone. Further, limitations for data on vascular density were that the sample sizes of study populations were relatively small; and that these studies did not account for a range of potential confounders (e.g. lifestyle factors).

### Section 3: Summary of human evidence on the associations of neuronal and microvascular structures and function of the neurovascular coupling unit, as quantified by retinal imaging, with cognitive performance

In this section, we review the present body of evidence on the associations of neuronal and microvascular structures and function of the neurovascular coupling unit, as quantified by retinal imaging, with cognitive performance. We searched for studies that assessed cognitive performance (not limited to a specific type of cognitive function) or cognitive status (defined as with dementia, mild cognitive impairment [MCI], or control) and retinal neurodegeneration (assessed as lower retinal nerve fiber layer [RNFL] thickness); retinal microvascular dysfunction (assessed as narrower or wider retinal arterioles and wider retinal venules); or retinal dysfunction of the neurovascular coupling unit (assessed as lower increase in retinal microvascular diameters in response to flicker light exposure [i.e. during an increase in neuronal activity]; lower perfusion of arterioles and venules at rest [assessed as lower velocity]; or lower capillary perfusion at rest[assessed as greater foveal avascular zone area or lower vascular density) (10, 17). Details on the search strategy are given in the **Supplemental Methods**.

#### Literature overview

**Table 3** shows the results of the literature search. We identified nine population-based studies that investigated the associations of RNFL thickness with cognitive performance (three studies had prospective data available [n=865-n=2,456] and all nine studies had cross-sectional data available [n=865-n=32,038]); and seven population-based studies that investigated the associations of retinal arteriolar and/or venular diameters with cognitive performance (one study was prospective [n=1,134] and six studies were cross-sectional [n=809-n=8,600]; an overview of smaller studies is given in the **Supplemental Table S4** as these studies have previously been reviewed) (81–87). In addition, we identified three studies that investigated the association of flicker light-induced increase in retinal microvascular diameters with cognitive performance (all cross-sectional studies; one population-based study [n=2,049] and two smaller clinical studies [n=54 and n=56]; all shown in **Table 3**). Also, we identified two studies that investigated the association of retinal arteriolar and/or venular perfusion at rest with cognitive performance (both cross-sectional; n=52 and n=17; both studies are shown in **Table 3**). We did not find any population-based studies with data on the associations of foveal avascular zone area or vascular density with cognitive performance. We did, however, find a recent meta-analysis (n=541) which showed the results of eight (non-population based) studies; and, in addition, we also found fourteen (non-population based) studies that were not considered for inclusion in the meta-analysis (as these studies were published after the last search date in the meta-analysis). **Table 3** shows the results of the meta-analysis as well as the results of the largest studies (studies with a study sample size N≥150) that were not included in this meta-analysis. An overview of the nine smaller studies is presented in **Supplemental Table S5**.

**Table 3:** Associations of neuronal and microvascular structures and function of the neurovascular coupling unit, as quantified by retinal imaging, with cognitive performance.

N	Cognitive performance assessment	Confounders	Main results	Reference
Retinal nerve fiber layer thickness				
*Prospective studies*				
N=1,251 (3-years follow-up)	Global cognitive performance was estimated from the following tests: prospective memory, pairs matching, numeric and verbal reasoning test, and reaction time.Cognitive decline was defined as an increased number of attempts on the prospective memory test, an increased number of incorrect matches on pairs matching, a decrease in the numeric and verbal reasoning test scores, or a reaction time slowed by at least 100 msec.	Age, sex, education, height, intraocular pressure, race/ethnicity, socioeconomic deprivation	Each quintile lower macular RNFL thickness (versus the most thick quintile) was significantly associated with an 18% increased risk of cognitive decline (OR = 1.18; 95% CI [1.08, 1.29]; P = 0.001).	Ko, 2018 (57);
N=2,456 (3-years follow-up)	Global cognitive performance was estimated from the verbal fluency test, 15-word learning test, letter-digit substitution test, Stroop test, and Purdue pegboard test. In addition cognitive performance was estimated from the MMSE.	Age, sex, education, BMI, blood pressure, antihypertensive medication, total cholesterol, HDL, diabetes, smoking	Lower peripapillary RNFL thickness was not significantly associated with lower global cognitive performance over time (per SD lower, -0.007; 95% CI [-0.030, 0.017] SD; P > 0.05). Lower peripapillary RNFL thickness was not significantly associated with a lower MMSE score over time (per SD lower, -0.004; 95% CI [-0.040, 0.032] SD; P > 0.05).	Mutlu, 2018 (58);
N=865 (baseline measurements in childhood [age 7-11 years old] and follow -up measurement at age 45 years old)	Change in cognitive performance from childhood (average of cognitive performance at ages 7, 9, and 11 years) to adulthood at (cognitive performance assessed at age 45 years). From the Wechsler Intelligence Scale for Children–Revised test and the Wechsler Adult Intelligence Scale test the following cognitive domains were assessed: Full Scale IQ, verbal comprehension, perceptual reasoning, and processing speed.	Sex, intraocular pressure, axial length, and optic disc area	Lower RNFL thickness was significantly associated with a reduction in processing speed from childhood to adulthood, but not with a change in global cognitive performance (estimated with the Full Scale IQ), verbal comprehension or perceptual reasoning from childhood to adulthood.	Barrett-Young, 2022 (59);
*Cross-sectional studies*				
N=32,038	Global cognitive performance was estimated from the following tests: prospective memory, pairs matching, numeric and verbal reasoning test, and reaction time.Test failure was defined as an incorrect answer on the first attempt of prospective memory, or doing worse than 95% of participants in pairs matching (>2 incorrect matches), numeric and verbal reasoning tests (score <3), or reaction time (>770 ms).	Age, sex, education, height, intraocular pressure, race/ethnicity, socioeconomic deprivation	The thinnest RNFL quintile (RNFL ≤ 45.9 µm) was 11% more likely to fail ≥ 1 cognitive test compared to the thickest RNFL (≥ 60.2 µm) quintile (OR = 1.11; 95% CI [1.02, 1.21]; P = 0.01).	Ko, 2018 (57);
N=5,347	MMSE, Boston Naming Test, Phonemic fluency, Semantic fluency animals, Trail Making Tests, Visuoconstruction copy, Visuoconstruction delayed recall, Wordlist learning, Wordlist delayed recall and Wordlist recognition, Reading the Mind in the Eyes test, Multiple choice German vocabulary test, and the Stroop-Test.	Age, sex, radius, education	Semantic fluency, Stroop test, and the Trail Making Tests (but not other tests) were significantly associated with peripapillary RNFL thickness.	Girbardt, 2021 (60);
*N= 5,563*	Short-form Mini-Mental State Examination, an animal naming task, a letter cancellation task, the Hopkins Verbal Learning Test, the National Adult Reading Test, and the Cambridge Neuropsychological Test Automated Battery Paired Associates Learning Test.	Disc area, age, sex, educational level, social class, visual acuity of the better eye, axial length, and a history of cataract surgery	A thicker RNFL thickness was associated with better scores for the short-form MMSE (per SD 0.06; 95% confidence interval [CI], [0.02, 0.10], *P* = 0.005), Hopkins Verbal Learning Test (0.16, 95% CI [0.03, 0.29]; *P* = 0.014), and The National Adult Reading Test (−0.24, 95% CI [−0.46, −0.02], *P* = 0.035). RNFL thickness was not associated with other cognitive indices.	Khawaja, 2016 (61);
*N=2,483*	Participants completed a 50-minute cognitive test battery that assessed 5 core cognitive domains across multiple modalities. To calculate global cognitive performance results of tests for subdomains were combined. The following domains were assessed. Executive function was assessed with a verbal fluency test (total number of animals named in 60 seconds), Trail-Making Test Part B (time to completion), and an eye-tracker anti-saccade task (percentage of erroneous prosaccades). Processing speed was assessed with Trail-Making Test Part A (time to completion) and an eye-tracker prosaccade task (mean latency). Working memory was assessed with a Corsi block tapping test (sum of forward and backward span) and a digit span test (sum of forward and backward span). Verbal episodic memory was assessed with 2 components of a verbal learning and memory test (immediate recall trials 1–5 total, delayed recall). Crystallized intelligence was assessed with a multiple-choice vocabulary intelligence test (total real words correctly identified).	Age, sex, education, spherical equivalent, visual acuity, smoking, diabetes, hypertension, history of stroke	Lower peripapillary RNFL thickness was not significantly associated with a lower global cognitive performance (per SD lower RNFL thickness = -0.021; 95% CI [-0.049, 0.007] SD; P> 0.05).	Ward, 2020 (62);
*N=3,243*	Global cognitive performance was estimated from the verbal fluency test, 15-word learning test, letter-digit substitution test, Stroop test, and Purdue pegboard test. In addition, cognitive performance was assessed with the MMSE.	Age, sex, education, BMI, blood pressure, antihypertensive medication, total cholesterol, HDL, diabetes, smoking	Lower peripapillary RNFL thickness was significantly associated with lower global cognitive performance (per SD lower, -0.044; 95% CI [-0.074, -0.013] SD; P < 0.05). Lower peripapillary RNFL thickness was significantly associated with a lower MMSE score (per SD lower, -0.056; 95% CI [-0.090, -0.022] SD; P < 0.05).	Mutlu, 2018 (58);
N=1,602	MMSE	Age, sex, education, socio-economic status, spherical equivalent, intraocular pressure, disc area	Lower macular RNFL thickness was significantly associated with a lower MMSE score (β = NR; 95% CI [NR]; P = 0.001).	Jones-Odeh, 2016 (63);
N=1,485	Different domains of cognitive functioning were assessed with the Dutch Adult Reading Test, the Rey Auditory Verbal Memory Test, semantic fluency, the Trail-Making Test, the Stroop Color-Word Test, and the Block Design test.	Age, sex, level of inbreeding, and refractive error	RNFL thickness was significantly associated with all tests of cognitive function, except for the Stroop Color-Word Test part III.	Van Koolwijk, 2009 (64);
N=865	Cognitive performance was assessed as the Full Scale IQ score at age 45 years.	Sex, intraocular pressure, axial length, and optic disc area	Lower RNFL thickness was significantly associated with a lower cognitive performance.	Barrett-Young, 2022 (59);
N=930 (patients from a memory clinic)	Cognitive status was assessed (N=414 cognitively healthy individuals; N=192 probable amnestic MCI; and N=324 probable AD)	Age, education, sex, and OCT image quality	No significant differences were observed between cognitively healthy individuals, individuals with probably amnestic MCI, or individuals with probable AD.	Sanchez, 2018 (65);
Retinal arteriolar diameter				
*Prospective studies*				
N=1,134 14-year follow-up.	Cognitive decline was defined as the 10% rapidest decline in test scores for the Delayed Word Recall Test (cut-off ≤−2.1/decade), Word Fluency Test (cut-off ≤−9.3/decade), and Digit Symbol Substitution Test (cut-off ≤−8.5/decade).	Age, sex, race, research center, hypertension, smoking, APOe4 status, diabetes	Individuals with generalized arteriolar narrowing (defined as the narrowest 25% of the population) versus individuals without generalized arteriolar narrowing did not have a greater cognitive decline based on the Delayed Word Recall Test (odds ratio [92%CI], 1.10 [0.63; 1.93]; the Word Fluency Test (1.00 [0.55; 1.80]), or the Digit Symbol Substitution Test (OR 0.95 [0.54-1.70]).	Lesage,2009 (66);
*Cross-sectional studies*				
N=1,988	Cognitive impairment assessed with the MMSE (cutoff for impairment MMSE score<23).	Age, sex, diabetes mellitus, smoking, post‐high school education, systolic blood pressure, and history of cardiovascular disease.	In individuals with hypertension, narrow versus wide retinal arteriolar diameter (defined as lowest quintile versus highest quintile) was not significantly associated with greater cognitive impairment (beta, lowest versus highest quintile, odds ratio 2.2 [0.9–5.2]). In individuals without hypertension, narrow versus wide retinal arteriolar diameter (defined as lowest quintile versus highest quintile) was not associated with greater cognitive impairment (beta, lowest versus highest quintile, odds ratio 0.4 [0.1–1.3]).	Liew, 2009 (67);
N=1,744	Executive function was assessed with the Digit symbol substitution test.	Age, sex, race, current smoking, heart disease, systolic blood pressure, natural logarithm of carotid intima-media thickness, and C-reactive protein; years of education, diabetes status, blocks walked per week, alcohol consumption; retinal venular diameter	Individuals with the narrowest retinal arteriolar diameter (narrowest 10% of the population versus the rest of the population) had a significantly lower score on the Digit symbol substitution test (beta, narrow versus wide, −1.26 [−2.39,−0.13] points).	Kim, 2011 (68);
N=8,600	Verbal learning and memory assessed with the delayed Word Recall Test (cut-off for low ≤4); executive function assessed with the Digit Symbol Subtest of the Wechsler Adult Intelligence Scale–Revised (cut-off for low ≤20); verbal fluency assessed with the Word Fluency Test of the Multilingual Aphasia Examination (cut-off for low ≤11).	Age, sex, race, research center, education, occupation, diabetes, fasting glucose, hypertension and mean arterial blood pressure averaged over visits 1 through 3, carotid intima-media thickness, cigarette smoking, alcohol consumption, fasting total and high density lipid cholesterol levels, and triglyceride levels.	Individuals with generalized arteriolar narrowing (defined as the narrowest 20% of the population based on the arteriolar-to-venular ratio), versus individuals without generalized arteriolar narrowing, did not have a lower score on the delayed word recall test (odds ratio [95%CI] 1.10 [0.80; 1.49]), the digit symbol subtest of the Wechsler Adult Intelligence Scale-Revised (odds ratio 1.08 [0.69-1.69]); or the Word Fluency Test of the Multilingual Aphasia Examination (odds ratio 1.37 [0.88; 2.12]).	Wong, 2002 (69);
N=2,211	Cognitive performance was assessed with the Modified Mini-Mental State Examination. Executive function was assessed with the Digit–Symbol Substitution Test.	Age, sex, race, field center, education level, internal carotid intima-media thickness, body mass index, hypertension, diabetes status, and cigarette smoking status	Test scores for Modified Mini-Mental State Examination or the Digit–Symbol Substitution Test did not differ between different quintiles of retinal arteriolar diameter.	Baker, 2007 (70);
N=954	Major domains of cognitive ability were assessed using tests of immediate and delayed non-verbal memory and verbal declarative memory (Faces and Family Pictures Subtest, Logical Memory subtest from the Wechsler Memory Scale-III); non-verbal reasoning, working memory, information processing speed (matrix reasoning, letter–number sequencing, Digit Symbol Test from the Wechsler Adult Intelligence Scale 3rd Edition); executive function (Borkowski Verbal Fluency Test); and mental flexibility (Trail Making Test-Part B). In addition, Vocabulary (‘crystallized’ intelligence) was measured (using the combined version of the Junior and Senior Mill Hill Vocabulary Scale synonyms) and global cognitive performance was assessed with the MMSE.	Age, sex, level of education, smoking, systolic blood pressure, major macrovascular disease and depression	Wider retinal arteriolar diameter was significantly associated with a lower memory test score in men, but not in women. In addition, there were no significant associations of retinal arteriolar diameter with other cognitive tests under study.	Ding, 2011 (71);
N=809	Cognitive screening was conducted during the in-home interview with the CASI-S, an abbreviated version of the Cognitive Abilities Screening Instrument The CASI-S includes four subtests: verbal registration (immediate recall of three words), temporal orientation, verbal fluency and verbal recall (delayed recall of three words). In a subset (n=281) cognitive performance was assessed with the SENAS test, which consists of the following measures: word list learning, spatial configuration learning, category fluency (animals & supermarket), phonemic fluency (letters F & L), list sorting (two lists), digit span forward, digit span backward, verbal conceptual thinking, object naming, picture association, pattern recognition and spatial localization that assess both verbal and non-verbal aspects of cognition	Age, sex, educational level, language of exam administration (Spanish, English), smoking, hypertension	Narrower retinal arteriolar diameters (continuous variable) and generalized arteriolar narrowing (defined as the lowest 25% of the data versus the rest of the data) were not statistically significantly associated with lower cognitive performance (estimated from the CASI-S test and the SENAS test).	Gatto, 2012 (72);
Retinal venular diameter				
*Prospective studies*				
No studies available				
*Cross-sectional studies*				
N=1,988	Cognitive impairment assessed with the MMSE (cutoff for impairment MMSE score<23).	Age, sex, diabetes mellitus, smoking, post‐high school education, systolic blood pressure, and history of cardiovascular disease.	In individuals with hypertension, wide versus narrow retinal venular diameter [defined as highest quartile of data versus other quartiles] was significantly associated with greater cognitive impairment (odds ratio 2.7 [1.2–6.1]). Alternatively, in individuals without hypertension, wide versus narrow retinal venular diameter was not associated with greater cognitive impairment (odds ratio 1.0 [0.4–2.4]).	Liew, 2009 (67);
N=1,744	Executive function was assessed with the Digit symbol substitution test.	Age, sex, race, current smoking, heart disease systolic blood pressure, natural logarithm of carotid intima-media thickness, and C-reactive protein; years of education, diabetes status, blocks walked per week, alcohol consumption; retinal arteriolar diameter	Individuals with the widest retinal venular diameter (widest 10% of the population versus the rest of the population) did not have a significantly lower score on the Digit symbol substitution test (1.35 [−0.12, 2.82]).	Kim, 2011 (68);
N=2,211	Cognitive function assessed with the Modified Mini-Mental State Examination Executive function was assessed with the Digit–Symbol Substitution Test.	Age, sex, race, field center, education level, internal carotid intima-media thickness, body mass index, hypertension, diabetes status, and cigarette smoking status.	Test scores for the Modified Mini-Mental State Examination or the Digit–Symbol Substitution Test did not differ between different quintiles of retinal venular diameter.	Baker, 2007 (70);
N=954	Major domains of cognitive ability were assessed using tests of immediate and delayed non-verbal memory and verbal declarative memory (Faces and Family Pictures Subtest, Logical Memory subtest from the Wechsler Memory Scale-III); non-verbal reasoning, working memory, information processing speed (matrix reasoning, letter–number sequencing, Digit Symbol Test from the Wechsler Adult Intelligence Scale 3rd Edition); executive function (Borkowski Verbal Fluency Test); and mental flexibility (Trail Making Test-Part B). In addition, Vocabulary (‘crystallized’ intelligence) was measured (using the combined version of the Junior and Senior Mill Hill Vocabulary Scale synonyms) and global cognitive performance was assessed with the MMSE.	Age, sex, level of education, smoking, systolic blood pressure, major macrovascular disease and depression	Wider retinal venular diameter was significantly associated with a lower memory test score in men, but not in women. In addition, there were no significant associations of retinal arteriolar diameter with other cognitive tests under study.	Ding, 2011 (71);
N=809	Cognitive screening was conducted during the in-home interview with the CASI-S, an abbreviated version of the Cognitive Abilities Screening Instrument The CASI-S includes four subtests: verbal registration (immediate recall of three words), temporal orientation, verbal fluency and verbal recall (delayed recall of three words). In a subset (n=281) cognitive performance was assessed with the SENAS test, which consists of the following measures: word list learning, spatial configuration learning, category fluency (animals & supermarket), phonemic fluency (letters F & L), list sorting (two lists), digit span forward, digit span backward, verbal conceptual thinking, object naming, picture association, pattern recognition and spatial localization that assess both verbal and non-verbal aspects of cognition	Age, sex, educational level, language of exam administration (Spanish, English), smoking, hypertension	A non-significant trend between generalized venular widening (defined as the highest 25% of the data versus the rest of the data) and lower cognitive performance was found (for both the CASI-S and the SENAS tests). However, wider retinal venular diameters, when analyzed as continuous measures, were not statistically significantly associated with lower cognitive performance (estimated from the CASI-S and SENAS tests).	Gatto, 2012 (72);
Flicker light-induced increase in retinal microvascular diameters				
*Prospective studies*				
No studies available				
*Cross-sectional studies*				
N=2,018 (arteriole); And N=2,049 (venule); Community-dwelling participants; oversampling of individuals with type 2 diabetes.	Global cognitive performance was calculated as the mean cognitive performance for the domains memory, information processing, and executive function. The composite memory score was derived from the Verbal Learning Test by weighting total immediate and delayed recall scores. The domain information processing speed included the Stroop Color-Word Test Part I and II, the Concept Shifting Test Part A and B, and the Letter Digit Substitution Test. Executive function was assessed by the Stroop Color-Word Test Part III and the Concept Shifting Test Part C.	age, education level, sex, type 2 diabetes, body mass index, smoking status, alcohol use, hypertension, total/high density lipid cholesterol ratio, triglycerides, lipid-modifying medication use, prior cardiovascular disease, current depression and plasma biomarkers of low-grade inflammation	Lower percentage flicker light-induced increase in retinal arteriolar or venular diameter was not significantly associated with lower cognitive performance (per SD lower arteriolar and venular percentage flicker light-induced increase [in SD], respectively, -0.016 [-0.050; 0.019]; and -0.022 [-0.056; 0.011]).	Rensma, 2019 (73);
N=56; N=12 individuals with AD; n= 12 individuals with MCI; and n=32 sex- and age-matched controls.	Diagnosis of AD and MCI was based on the National Institute on Aging-Alzheimer’s Association criteria.	None	The mean flicker light-induced percentage increase in retinal arteriolar diameter was significantly lower in individuals with AD (mean percentage increase: 0.21 ± 1.80%) and in individuals with MCI (mean percentage increase: 2.29 ± 1.81%) than in healthy controls (mean percentage increase: 3.86 ± 1.94%). The mean flicker light-induced percentage increase in retinal venular diameter was significantly lower in individuals with AD (2.84 ± 1.56; P<0.05) than in individuals with MCI (3.45 ± 1.96) or healthy controls (3.37 ± 1.81), however, there were no significant differences between individuals with MCI or healthy controls.	Querques, 2019 (74);
N=54; N=15 individuals with AD; n= 24 individuals with MCI; and n=15 healthy controls.	Diagnosis of AD and MCI was based on the National Institute on Aging-Alzheimer’s Association criteria.	None	The percentage mean maximal flicker light-induced increase in retinal arteriolar diameter was significantly greater in individuals with AD in comparison with healthy controls (P = 0.004), and numerically in between in individuals with MCI (though not statistically significantly differently from healthy controls). The percentage mean maximal flicker light-induced increase in retinal venular diameter was significantly greater in individuals with AD in comparison with healthy controls (P = 0.001), and numerically in between (though not statistically significant from healthy controls) in individuals with MCI.	Kotliar, 2017 (75);
Retinal blood flow				
*Prospective studies*				
No studies available				
*Cross-sectional studies*				
N=52; N=10 individuals with AD; n= 21 individuals with MCI; and n=21 healthy controls.	MCI diagnosis was based on the 2004 MCI Working Group Criteria for amnestic MCI and on the global clinical dementia rating. AD diagnosis was based on the Diagnostic and Statistical Manual of Mental Disorders, 4th Edition (DSM-IV) diagnostic criteria for dementia and National Institute of Neurological and Communicative Disorders and Stroke and the Alzheimer’s Disease and Related Disorders Association criteria.	None	In individuals with AD (mean venous blood speed 29.1 ± 6.3 mm/s) and MCI (mean venous blood speed 28.9 ± 6.3 mm/s) the venous blood speed was significantly lower than in healthy control individuals (mean venous blood speed 36.0 ± 9.3 mm/s) In individuals with AD (mean venous blood flow 12.3 ± 2.8 μl/min) and MCI (mean venous blood flow 16.1 ± 4.4 μl/min) the venous blood flow was significantly lower than in healthy control individuals (mean venous blood flow 20.0 ± 5.4 μl/min).	Feke, 2015 (76);
N=17; N=9 individuals with AD; and n=8 healthy controls.	AD diagnosis was based on the Diagnostic and Statistical Manual of Mental Disorders, 4th Edition (DSM-IV) diagnostic criteria for dementia and National Institute of Neurological and Communicative Disorders and Stroke and the Alzheimer’s Disease and Related Disorders Association criteria.	None	The venous blood flow rate was significantly lower in individuals with AD (9.7 ± 3.1 μL/min) than in healthy controls (15.9 ± 3.7 μL/min, P = 0.002). The venous centerline blood speed was lower, but not statistically significantly, in individuals with AD (23.8 ± 8.2 mm/s) than in healthy controls (30.8 ± 6.0 mm/s; *P* = 0.07).	Berisha, 2007 (77);
Vascular density and size of the foveal avascular area				
*Prospective studies*				
N=297; 10-years follow-up; Community-dwelling participants	A 10-test neurocognitive battery spanning several cognitive domains (memory and speed of processing/executive function) was administered. Diagnosis of AD and MCI was based on the National Institute on Aging-Alzheimer’s Association criteria.	age, income, education, race/community (black/Jackson or white/Washington Co), current smoking, physical activity, hypertension, and diabetes.	Greater superficial vascular complex density, greater deep capillary plexus vessel density and greater foveal avascular zone area were not significantly associated with change in global cognitive performance (per 10% increase in superficial vascular complex density or deep capillary plexus vessel density or per 1 mm^2^ larger foveal avascular zone area, respectively, 0.04 [−0.07, 0.15]; 0.03 [−0.08, 0.14], and 0.24 [−0.35, 0.83] SD).	Abraham, 2021 (78);
*Cross-sectional studies*				
N=571 (Meta-analysis; N=150 individuals with AD; n= 195 individuals with MCI; and n=226 healthy controls	AD and MCI were defined in individual studies.	None	Individuals with MCI, versus healthy controls, had a significantly lower vessel density in the superficial capillary plexus: weighted mean difference: -2.26, 95% CI: -3.98 to -0.55, P = 0.01; in the deep capillary plexus: weighted mean difference: -3.40, 95% CI: -5.99 to -0.81, P = 0.01; and a larger fovea avascular zone area: weighted mean difference = 0.06, 95% CI: 0.01 to 0.11, P = 0.02. Individuals with AD, versus healthy controls, had a significantly lower vessel density in the superficial capillary plexus: weighted mean difference = -1.88, 95% CI: -2.7 to -1.07, p<0.00001. In contrast, there was no significant decrease in the deep capillary plexus nor enlargement of the avascular zone in individuals with AD.	Hui, 2021 (79);
N=297; Community-dwelling participants	A 10-test neurocognitive battery spanning several cognitive domains (memory and speed of processing/executive function) was administered. Diagnosis of AD and MCI was based on the National Institute on Aging-Alzheimer’s Association criteria.	Age, sex, income, education, race/community (black/Jackson or white/Washington Co), current smoking, physical activity, hypertension, and diabetes	Greater superficial vascular complex density, greater deep capillary plexus vessel density and greater foveal avascular zone area were not associated with global cognitive performance (per 10% increase in superficial vascular complex density or deep capillary plexus vessel density or per 1 mm^2^ larger foveal avascular zone area, respectively, −0.02 [−0.7, 0.01]; −0.01 [−0.06, 0.09] and 0.23 [−0.31, 0.77] SD).	Abraham, 2021 (78);
N=177; Chronic kidney disease stage ≥3 (including end-stage renal disease).	MMSE	The following variables were combined in 1 model: RNFL thickness, ganglion complex cell thickness, superficial vascular complex density, and deep capillary plexus vessel density, age, sex, estimated glomerular filtration rate, diabetes mellitus, hypertension, dyslipidemia, and years of education	Greater superficial vascular complex density was not associated with global cognitive performance but greater deep capillary plexus vessel density was (per percentage greater superficial vascular complex density and deep capillary plexus vessel density, respectively, -0.07 [-0.16; 0.01] and 0.11 [0.01; 0.21]).	Peng, 2021 (80);

**Table 3** shows the associations of neuronal and microvascular structures and function of the neurovascular coupling unit, as quantified by retinal imaging, with cognitive performance. Fully adjusted results are shown (where appropriate, i.e. when associations where adjusted for confounders).

MMSE, Mini-Mental State Examination; RNFL, retinal nerve fiber layer thickness; OR, odds ratio; SD, standard deviation; CI, confidence interval; MCI, mild cognitive impairment; AD, Alzheimer’s disease.

#### Summary of findings

Most population-based studies found that lower RNFL thickness, narrower retinal arteriolar diameters, and wider retinal venular diameters were associated with lower cognitive performance. In addition, most small studies found that lower flicker light-induced increase in retinal microvascular diameters, lower retinal arteriolar and venular perfusion at rest, lower vascular density, and greater foveal avascular zone area were associated with lower cognitive performance. These findings are consistent with the concept that retinal imaging techniques may provide an opportunity to study the early pathobiology of cognitive dysfunction.

#### Limitations

The present body of evidence has certain limitations. The main limitation of the available population-based data on the associations of RNFL thickness and retinal microvascular diameters with cognitive performance is that most studies were cross-sectional in design (8). In addition, population-based data were lacking for associations of flicker light-induced increase in retinal microvascular diameters, retinal arteriolar and venular perfusion at rest, and capillary perfusion at rest, estimated from vascular density and the size of the foveal avascular zone, with cognitive performance; and most studies did not account for a range of potential confounders (e.g. lifestyle factors) (8).

## Discussion

This review has two main results. First, we found that there is limited data on the association between dysfunction of the neurovascular coupling unit, as quantified with *cerebral* imaging techniques, and cognitive dysfunction. Second, we found that neurodegeneration, microvascular dysfunction, and dysfunction of the neurovascular coupling unit, as quantified with *retinal* imaging techniques, are associated with a higher prevalence of MRI features of structural brain abnormalities and lower cognitive performance. Retinal imaging techniques are more easily applied at large scale as compared to cerebral imaging techniques and, thus, may provide an opportunity to study the early pathobiology of dysfunction of the neurovascular coupling unit and cognitive dysfunction. Below we discuss advantages and disadvantages of this proposal and highlight directions for future research.

Advantages of retinal imaging techniques are that these techniques are non-invasive, relatively inexpensive, highly accurate, require relatively short measurement time, and that the biological interpretation of findings is relatively clear. Indeed, neuronal and microvascular structures can be separately assessed and different levels of the microcirculation can be separately assessed (i.e. arterioles, venules, and capillaries) (10).

A potential disadvantage of studying neurovascular coupling at the level of the retina is that is it not possible to study regional differences in the brain (10). Although neurovascular dysfunction may be a generalized phenomenon in the central nervous system, possibly the pathophysiological mechanisms that underlie degeneration of the neurovascular coupling unit may differ between different brain regions (10, 42, 88). Another potential disadvantage is that no direct estimate of blood-retina barrier integrity is available, although structural indices of capillary density as estimated with OCT-A may be related to blood-retina barrier integrity (10, 42, 88). However, this requires further study (10, 42, 88).

Future research is necessary to move towards a better under understanding of dysfunction of the neurovascular coupling unit and, ultimately, the development of early therapeutic strategies (based on the early modification of adverse risk factors such as hyperglycemia) to prevent dysfunction of the neurovascular coupling unit. Future research is needed to evaluate how and to what extent neurodegeneration and microvascular dysfunction, and loss of their interaction, contribute to dysfunction of the neurovascular coupling unit (6, 7, 11–13). As presumedly a bi-directional interaction exists between neuronal and microvascular structures, individuals with both degeneration of neuronal and microvascular structures may be at an increased risk for cognitive decline and dementia. In addition, it is important that future studies quantify how potentially modifiable risk factors are related to loss or dysfunction of the individual components of the neurovascular coupling unit; and investigate whether targeted modification of these risk factors may reduce degeneration of neuronal and microvascular structures, dysfunction of the neurovascular coupling unit, and, can thereby, delay and/or prevent the onset of dementia (2). Further, studies are warranted to investigate whether retinal imaging techniques that assess deterioration of components of the neurovascular coupling unit (i.e. neuronal and microvascular structures) and function of the neurovascular coupling unit may be means for the early stage identification of individuals at risk for cognitive decline and dementia.

An important strength of the present review is that we provided a complete and extensive overview of the presently available population-based data (8). We reported data from many large studies, data which we presume to be relatively of a lower risk of selection bias than smaller clinical studies; and these studies accounted for a large number of potential confounders, which reduces the chance that the results of these studies are spuriously estimated due to residual confounding (8).

This review has certain limitations. To start, as the review reports extensively on findings in the general population, some matters have not been addressed in great detail. We did not detail: 1) findings in subgroups stratified by key characteristics (e.g. age, sex, ethnicity, or apolipoprotein E status); 2) how differences in the classification of mild cognitive impairment or dementia between studies may have affected the results; and 3) exclusion criteria used in different studies (e.g. in some, but not all, studies individuals with eye diseases such as glaucoma and diabetic retinopathy were excluded).

Next, we made certain assumptions, which should be reported as potential limitations: 1) we defined population-based studies as those with sample sizes of n=500 or more assuming this would constitute a representative sample (8); and 2) we presumed that SPECT-, ASL-MRI-, and BOLD fMRI-assessed cerebral blood flow and/or cerebrovascular reactivity reflect function of the neurovascular coupling unit. However, with regard to the latter, this assumption should be made with caution as these measures are indirect, and not direct, measures of the function of the neurovascular coupling unit. We presume that these measures reflect function of the neurovascular coupling unit because cerebral blood flow and cerebrovascular reactivity are to an important extent regulated by the neurovascular coupling unit (14).

## Conclusion

This review highlights that 1) the association between dysfunction of the neurovascular coupling unit, assessed in the brain, and early stage cognitive dysfunction remains incompletely understood as population-based data are lacking; and 2) that studying the retina may provide opportunity to acquire population-based data on the association between dysfunction of the neurovascular coupling unit and early-stage cognitive dysfunction, including cerebral small vessel disease. A better understanding of the early pathobiology of cognitive dysfunction may contribute to the development of novel therapeutic strategies that aim to prevent dementia.

## Author contributions

FH contributed to conception and design, participated in acquisition of data, analyzed and interpreted data, and drafted the manuscript. NW participated in acquisition of data. TS contributed to conception and design of the study and interpreted the data. All authors revised the manuscript critically for important intellectual content, and provided final approval of the version to be published. FH is the guarantor of this work and, as such, takes responsibility for the integrity of the data and the accuracy of the data analysis. All authors contributed to the article and approved the submitted version.

## Funding

TS is supported by a VENI research grant (916.19.074) from The Netherlands Organization for Scientific Research (NWO) and The Netherlands Organization for Health Research and Development (ZonMw), a Dutch Heart Foundation research grant (2018T025), and a Junior Fellowship from the Dutch Diabetes Research Foundation.

## Conflict of interest

The authors declare that the research was conducted in the absence of any commercial or financial relationships that could be construed as a potential conflict of interest.

## Publisher’s note

All claims expressed in this article are solely those of the authors and do not necessarily represent those of their affiliated organizations, or those of the publisher, the editors and the reviewers. Any product that may be evaluated in this article, or claim that may be made by its manufacturer, is not guaranteed or endorsed by the publisher.
