# Supplementary material for: Neurovascular coupling unit dysfunction and dementia: Retinal measurements as tools to move towards population-based evidence
Source: Front Endocrinol (Lausanne). 2022 Nov 23;13:1014287. doi: 10.3389/fendo.2022.1014287 (PMC9727310; doi:10.3389/fendo.2022.1014287)
Supplement: Supplementary file 1 [file DataSheet_1.docx]

### **Supplemental Material**

Content

Supplemental Methods

Supplemental Results

**Supplemental Methods**

We searched PubMed up to the 31^st^ of January 2022 to identify studies. Search terms are shown in Supplemental Table S1. We combined search terms using “OR” (for search terms which are related to the same key word) or “AND” (to combine multiple different key words in to a search string). We did not include studies that used certain specific study populations (e.g. individuals with Parkinson’s disease; multiple sclerosis; or migraine).

Supplemental Table S1 Search terms used to identify relevant literature.

| Key word | Search terms |
| --- | --- |
| Cognitive performance or cognitive status | Cognitive performance; cognitive function; memory; executive function; information processing speed; mild cognitive impairment; MCI; Alzheimer’s disease; AD. |
| Brain imaging tools for the assessment of the function of the neurovascular coupling unit * | Single-photon emission computed tomography; SPECT; blood oxygen level-dependent; BOLD; arterial spin labelling; ASL; dynamic contrast enhanced; DCE; CSF/plasma-albumin ratio; Cerebral spinal fluid plasma albumin ratio. |
| Retinal microvascular diameters and retinal nerve fiber layer thickness | Central retinal arteriolar equivalent; CRAE; central retinal venular equivalent; CRVE; arteriole-to-venule ratio; AVR; retinal microvascular diameters; retinal calibers; retinal nerve fiber layer thickness; retinal nerve fibre layer thickness; RNFL; optical coherence tomography. |
| Brain imaging tools for the assessment of the function of the neurovascular coupling unit in the retina | Dynamic vessel analyzer; DVA, flicker light-induced increase in retinal microvascular diameters; flicker light-induced increase in retinal arteriolar diameter; flicker light-induced increase in retinal venular diameter; retinal laser Doppler flowmetry; retinal flowmetry; optical coherence tomography angiography; OCT-A; OCT angiography. |

Supplemental Table S1 shows search terms used to identify relevant literature.

* As our aim was to gain insight in the early pathobiology of cognitive dysfunction, we limited the evidence overview to studies with data from (mainly) individuals with mild cognitive impairment (MCI) or with normal cognitive function (i.e. without cognitive impairment). To limit our search for ASL-MRI to the early-stage of cognitive dysfunction and MCI, we added “cognitively unimpaired” to the search.

**Supplemental Results**

*Search for ASL-MRI and cognitive performance or cognitive status (Table 1)*

We show findings for studies with a study population N≥150 in Table 1. Supplemental Table S2 shows the studies with smaller study populations (which were omitted from Table 1).

*Search for retinal imaging techniques and cerebral MRI features of structural brain abnormalities (Table 2)*

We show findings for studies with a study population N≥500 in Table 2. Supplemental Table S3 shows the studies with smaller study populations (which were omitted from Table 2).

*Search for RNFL thickness and retinal microvascular diameters and cognitive performance or cognitive status (Table 3)*

We show findings for studies with a study population N≥500 in Table 3. Supplemental Table S4 shows the studies with smaller study populations (which were omitted from Table 3).

*Search for OCT-A and cognitive performance or cognitive status (Table 3)*

In Table 3 we show findings of a meta-analysis and, in addition, findings of the largest studies (with a study population N≥150) that were published after the last literature search date in this meta-analysis. Supplemental Table S5 shows the studies with smaller study populations (with a study population N<150) that were published after the last literature search date in the meta-analysis (these studies were omitted from Table 3).

Supplemental Table S2 Studies omitted from the literature overview on ASL-MRI and cognitive performance or cognitive status

| Study population size N<150 |
| --- |
| Huang H, Zhao K, Zhu W, Li H, Zhu W. Abnormal Cerebral Blood Flow and Functional Connectivity Strength in Subjects With White Matter Hyperintensities. Front Neurol. 2021 Oct 20;12:752762. doi: 10.3389/fneur.2021.752762. PMID: 34744987; PMCID: PMC8564178. |
| Duan W, Zhou GD, Balachandrasekaran A, Bhumkar AB, Boraste PB, Becker JT, Kuller LH, Lopez OL, Gach HM, Dai W. Cerebral Blood Flow Predicts Conversion of Mild Cognitive Impairment into Alzheimer's Disease and Cognitive Decline: An Arterial Spin Labeling Follow-up Study. J Alzheimers Dis. 2021;82(1):293-305. doi: 10.3233/JAD-210199. PMID: 34024834; PMCID: PMC8527573. |
| Bangen KJ, Thomas KR, Sanchez DL, Edmonds EC, Weigand AJ, Delano-Wood L, Bondi MW; Alzheimer’s Disease Neuroimaging Initiative. Entorhinal Perfusion Predicts Future Memory Decline, Neurodegeneration, and White Matter Hyperintensity Progression in Older Adults. J Alzheimers Dis. 2021;81(4):1711-1725. doi: 10.3233/JAD-201474. PMID: 33967041. |
| Li P, Mu J, Ma X, Ding D, Ma S, Zhang H, Liu J, Zhang M. Neurovascular coupling dysfunction in end-stage renal disease patients related to cognitive impairment. J Cereb Blood Flow Metab. 2021 Oct;41(10):2593-2606. doi: 10.1177/0271678X211007960. Epub 2021 Apr 14. PMID: 33853410; PMCID: PMC8504946. |
| Duan W, Sehrawat P, Balachandrasekaran A, Bhumkar AB, Boraste PB, Becker JT, Kuller LH, Lopez OL, Gach HM, Dai W. Cerebral Blood Flow Is Associated with Diagnostic Class and Cognitive Decline in Alzheimer's Disease. J Alzheimers Dis. 2020;76(3):1103-1120. doi: 10.3233/JAD-200034. PMID: 32597803; PMCID: PMC7970411. |
| Chau ACM, Cheung EYW, Chan KH, Chow WS, Shea YF, Chiu PKC, Mak HKF. Impaired cerebral blood flow in type 2 diabetes mellitus - A comparative study with subjective cognitive decline, vascular dementia and Alzheimer's disease subjects. Neuroimage Clin. 2020;27:102302. doi: 10.1016/j.nicl.2020.102302. Epub 2020 May 28. PMID: 32521474; PMCID: PMC7284123 |
| Wang X, Ding D, Zhao Q, Liang X, Peng L, Zhao X, Xi Q, Min Z, Wang W, Xu X, Guo Q, Wang PJ. Brain hemodynamic changes in amnestic mild cognitive impairment measured by pulsed arterial spin labeling. Aging (Albany NY). 2020 Mar 12;12(5):4348-4356. doi: 10.18632/aging.102888. Epub 2020 Mar 12. PMID: 32167487; PMCID: PMC7093201 |
| Hays CC, Zlatar ZZ, Meloy MJ, Bondi MW, Gilbert PE, Liu T, Helm JL, Wierenga CE. Interaction of APOE, cerebral blood flow, and cortical thickness in the entorhinal cortex predicts memory decline. Brain Imaging Behav. 2020 Apr;14(2):369-382. doi: 10.1007/s11682-019-00245-x. PMID: 32048144; PMCID: PMC7165062. |
| Dolui S, Li Z, Nasrallah IM, Detre JA, Wolk DA. Arterial spin labeling versus 18F-FDG-PET to identify mild cognitive impairment. Neuroimage Clin. 2020;25:102146. doi: 10.1016/j.nicl.2019.102146. Epub 2019 Dec 23. PMID: 31931403; PMCID: PMC6957781. |
| Wang J, Peng G, Liu P, Tan X, Luo B; Alzheimer’s Disease Neuroimaging Initiative. Regulating effect of CBF on memory in cognitively normal older adults with different ApoE genotype: the Alzheimer's Disease Neuroimaging Initiative (ADNI). Cogn Neurodyn. 2019 Dec;13(6):513-518. doi: 10.1007/s11571-019-09536-x. Epub 2019 May 11. PMID: 31741688; PMCID: PMC6825072. |
| Yu Y, Yan LF, Sun Q, Hu B, Zhang J, Yang Y, Dai YJ, Cui WX, Xiu SJ, Hu YC, Heng CN, Liu QQ, Hou JF, Pan YY, Zhai LH, Han TH, Cui GB, Wang W. Neurovascular decoupling in type 2 diabetes mellitus without mild cognitive impairment: Potential biomarker for early cognitive impairment. Neuroimage. 2019 Oct 15;200:644-658. doi: 10.1016/j.neuroimage.2019.06.058. Epub 2019 Jun 25. PMID: 31252056. |
| Huang Q, Cao X, Chai X, Wang X, Xu L, Xiao C. Three-dimensional pseudocontinuous arterial spin labeling and susceptibility-weighted imaging associated with clinical progression in amnestic mild cognitive impairment and Alzheimer's disease. Medicine (Baltimore). 2019 Jun;98(23):e15972. doi: 10.1097/MD.0000000000015972. PMID: 31169728; PMCID: PMC6571427. |
| Hu B, Yan LF, Sun Q, Yu Y, Zhang J, Dai YJ, Yang Y, Hu YC, Nan HY, Zhang X, Heng CN, Hou JF, Liu QQ, Shao CH, Li F, Zhou KX, Guo H, Cui GB, Wang W. Disturbed neurovascular coupling in type 2 diabetes mellitus patients: Evidence from a comprehensive fMRI analysis. Neuroimage Clin. 2019;22:101802. doi: 10.1016/j.nicl.2019.101802. Epub 2019 Mar 27. PMID: 30991623; PMCID: PMC6447740. |
| Xu Y, Chen LL, Su Y, Sun SN, Zhang HY. [Regression analysis of cerebral blood perfusion and cognitive function in patients with mild cognitive impairment and Alzheimer's disease]. Zhonghua Yi Xue Za Zhi. 2019 Jan 15;99(3):193-197. Chinese. doi: 10.3760/cma.j.issn.0376-2491.2019.03.008. PMID: 30669762. |
| Cheng BC, Chen PC, Chen PC, Lu CH, Huang YC, Chou KH, Li SH, Lin AN, Lin WC. Decreased cerebral blood flow and improved cognitive function in patients with end-stage renal disease after peritoneal dialysis: An arterial spin-labelling study. Eur Radiol. 2019 Mar;29(3):1415-1424. doi: 10.1007/s00330-018-5675-9. Epub 2018 Aug 13. PMID: 30105409; PMCID: PMC6510858. |
| Riederer I, Bohn KP, Preibisch C, Wiedemann E, Zimmer C, Alexopoulos P, Förster S. Alzheimer Disease and Mild Cognitive Impairment: Integrated Pulsed Arterial Spin-Labeling MRI and 18F-FDG PET. Radiology. 2018 Jul;288(1):198-206. doi: 10.1148/radiol.2018170575. Epub 2018 May 15. PMID: 29762090. |
| Rane S, Koh N, Boord P, Madhyastha T, Askren MK, Jayadev S, Cholerton B, Larson E, Grabowski TJ. Quantitative cerebrovascular pathology in a community-based cohort of older adults. Neurobiol Aging. 2018 May;65:77-85. doi: 10.1016/j.neurobiolaging.2018.01.006. Epub 2018 Jan 31. PMID: 29452984; PMCID: PMC5871567 |
| Lassila T, Di Marco LY, Mitolo M, Iaia V, Levedianos G, Venneri A, Frangi AF. Screening for Cognitive Impairment by Model-Assisted Cerebral Blood Flow Estimation. IEEE Trans Biomed Eng. 2018 Jul;65(7):1654-1661. doi: 10.1109/TBME.2017.2759511. Epub 2017 Oct 5. PMID: 28991728. |
| Dolui S, Vidorreta M, Wang Z, Nasrallah IM, Alavi A, Wolk DA, Detre JA. Comparison of PASL, PCASL, and background-suppressed 3D PCASL in mild cognitive impairment. Hum Brain Mapp. 2017 Oct;38(10):5260-5273. doi: 10.1002/hbm.23732. Epub 2017 Jul 24. PMID: 28737289; PMCID: PMC5593784. |
| Cui Y, Liang X, Gu H, Hu Y, Zhao Z, Yang XY, Qian C, Yang Y, Teng GJ. Cerebral perfusion alterations in type 2 diabetes and its relation to insulin resistance and cognitive dysfunction. Brain Imaging Behav. 2017 Oct;11(5):1248-1257. doi: 10.1007/s11682-016-9583-9. PMID: 27714551; PMCID: PMC5653700. |
| Jiang XL, Wen JQ, Zhang LJ, Zheng G, Li X, Zhang Z, Liu Y, Zheng LJ, Wu L, Chen HJ, Kong X, Luo S, Lu GM, Ji XM, Zhang ZJ. Cerebral blood flow changes in hemodialysis and peritoneal dialysis patients: an arterial-spin labeling MR imaging. Metab Brain Dis. 2016 Aug;31(4):929-36. doi: 10.1007/s11011-016-9829-7. Epub 2016 May 11. PMID: 27167984. |
| Nicholas CR, Okonkwo OC, Bendlin BB, Oh JM, Asthana S, Rowley HA, Hermann B, Sager MA, Johnson SC. Posteromedial hyperactivation during episodic recognition among people with memory decline: findings from the WRAP study. Brain Imaging Behav. 2015 Dec;9(4):690-702. doi: 10.1007/s11682-014-9322-z. PMID: 25332108; PMCID: PMC4405422. |
| Alosco ML, Gunstad J, Beard C, Xu X, Clark US, Labbe DR, Jerskey BA, Ladino M, Cote DM, Walsh EG, Poppas A, Cohen RA, Sweet LH. The synergistic effects of anxiety and cerebral hypoperfusion on cognitive dysfunction in older adults with cardiovascular disease. J Geriatr Psychiatry Neurol. 2015 Mar;28(1):57-66. doi: 10.1177/0891988714541871. Epub 2014 Jul 9. PMID: 25009160; PMCID: PMC4289129. |
| Ding B, Ling HW, Zhang Y, Huang J, Zhang H, Wang T, Yan FH. Pattern of cerebral hyperperfusion in Alzheimer's disease and amnestic mild cognitive impairment using voxel-based analysis of 3D arterial spin-labeling imaging: initial experience. Clin Interv Aging. 2014 Mar 26;9:493-500. doi: 10.2147/CIA.S58879. PMID: 24707173; PMCID: PMC3971940. |
| Zhang Q, Stafford RB, Wang Z, Arnold SE, Wolk DA, Detre JA. Microvascular perfusion based on arterial spin labeled perfusion MRI as a measure of vascular risk in Alzheimer's disease. J Alzheimers Dis. 2012;32(3):677-87. doi: 10.3233/JAD-2012-120964. PMID: 22886015; PMCID: PMC3646066. |
| Wierenga CE, Dev SI, Shin DD, Clark LR, Bangen KJ, Jak AJ, Rissman RA, Liu TT, Salmon DP, Bondi MW. Effect of mild cognitive impairment and APOE genotype on resting cerebral blood flow and its association with cognition. J Cereb Blood Flow Metab. 2012 Aug;32(8):1589-99. doi: 10.1038/jcbfm.2012.58. Epub 2012 May 2. PMID: 22549621; PMCID: PMC3421098. |
| Bangen KJ, Restom K, Liu TT, Wierenga CE, Jak AJ, Salmon DP, Bondi MW. Assessment of Alzheimer's disease risk with functional magnetic resonance imaging: an arterial spin labeling study. J Alzheimers Dis. 2012;31 Suppl 3(0):S59-74. doi: 10.3233/JAD-2012-120292. PMID: 22531427; PMCID: PMC3443702. |
| Alexopoulos P, Sorg C, Förschler A, Grimmer T, Skokou M, Wohlschläger A, Perneczky R, Zimmer C, Kurz A, Preibisch C. Perfusion abnormalities in mild cognitive impairment and mild dementia in Alzheimer's disease measured by pulsed arterial spin labeling MRI. Eur Arch Psychiatry Clin Neurosci. 2012 Feb;262(1):69-77. doi: 10.1007/s00406-011-0226-2. Epub 2011 Jul 24. PMID: 21786091. |

Supplemental Table S2 shows an overview of studies that were omitted from the literature overview on ASL-MRI and cognitive performance or cognitive status in Table 1.

Abbreviations: ASL-MRI, arterial spin labelling magnetic resonance imaging.

Supplemental Table S3 Studies omitted from the literature overview for associations of neuronal and microvascular structures and function of the neurovascular coupling unit, as quantified by retinal imaging, with cerebral MRI features of structural brain abnormalities

| Study population size N=300-499 |
| --- |
| Ikram MK, De Jong FJ, Van Dijk EJ, Prins ND, Hofman A, Breteler MM, De Jong PT. Retinal vessel diameters and cerebral small vessel disease: the Rotterdam Scan Study. Brain. 2006 Jan;129(Pt 1):182-8. doi: 10.1093/brain/awh688. Epub 2005 Nov 29. PMID: 16317022. |
| Study population size N<300 |
| Qu M, Kwapong WR, Peng C, Cao Y, Lu F, Shen M, Han Z. Retinal sublayer defect is independently associated with the severity of hypertensive white matter hyperintensity. Brain Behav. 2020 Feb;10(2):e01521. doi: 10.1002/brb3.1521. Epub 2019 Dec 25. PMID: 31875660; PMCID: PMC7010590. |
| Shi Z, Zheng H, Hu J, Jiang L, Cao X, Chen Y, Mei X, Li C, Shen Y. Retinal Nerve Fiber Layer Thinning Is Associated With Brain Atrophy: A Longitudinal Study in Nondemented Older Adults. Front Aging Neurosci. 2019 Apr 11;11:69. doi: 10.3389/fnagi.2019.00069. PMID: 31031615; PMCID: PMC6470389. |
| Peng C, Kwapong WR, Xu S, Muse FM, Yan J, Qu M, Cao Y, Miao H, Zhen Z, Wu B, Han Z. Structural and Microvascular Changes in the Macular Are Associated With Severity of White Matter Lesions. Front Neurol. 2020 Jun 30;11:521. doi: 10.3389/fneur.2020.00521. PMID: 32714262; PMCID: PMC7344221. |
| Donix M, Wittig D, Hermann W, Haussmann R, Dittmer M, Bienert F, Buthut M, Jacobi L, Werner A, Linn J, Ziemssen T, Brandt MD. Relation of retinal and hippocampal thickness in patients with amnestic mild cognitive impairment and healthy controls. Brain Behav. 2021 May;11(5):e02035. doi: 10.1002/brb3.2035. Epub 2021 Jan 15. PMID: 33448670; PMCID: PMC8119792. |
| Doubal FN, MacGillivray TJ, Hokke PE, Dhillon B, Dennis MS, Wardlaw JM. Differences in retinal vessels support a distinct vasculopathy causing lacunar stroke. Neurology. 2009 May 19;72(20):1773-8. doi: 10.1212/WNL.0b013e3181a60a71. PMID: 19451533; PMCID: PMC2827311. |
| Hilal S, Ong YT, Cheung CY, Tan CS, Venketasubramanian N, Niessen WJ, Vrooman H, Anuar AR, Chew M, Chen C, Wong TY, Ikram MK. Microvascular network alterations in retina of subjects with cerebral small vessel disease. Neurosci Lett. 2014 Aug 8;577:95-100. doi: 10.1016/j.neulet.2014.06.024. Epub 2014 Jun 14. PMID: 24937268. |
| Zhang Y, Zhang Z, Zhang M, Cao Y, Yun W. Correlation Between Retinal Microvascular Abnormalities and Total Magnetic Resonance Imaging Burden of Cerebral Small Vessel Disease in Patients With Type 2 Diabetes. Front Neurosci. 2021 Dec 14;15:727998. doi: 10.3389/fnins.2021.727998. PMID: 34970109; PMCID: PMC8712683. |
| Huang KK, Huang S, Yun WW, Zhang ZX, Jia YW, Zhang M. [Correlation between total cerebral small vessel disease score and retinal vessel diameters in patients with mild stroke]. Zhonghua Yi Xue Za Zhi. 2021 Jan 5;101(1):62-67. Chinese. doi: 10.3760/cma.j.cn112137-20200405-01088. PMID: 33423447. |
| Jung NY, Han JC, Ong YT, Cheung CY, Chen CP, Wong TY, Kim HJ, Kim YJ, Lee J, Lee JS, Jang YK, Kee C, Lee KH, Kim EJ, Seo SW, Na DL. Retinal microvasculature changes in amyloid-negative subcortical vascular cognitive impairment compared to amyloid-positive Alzheimer's disease. J Neurol Sci. 2019 Jan 15;396:94-101. doi: 10.1016/j.jns.2018.10.025. Epub 2018 Oct 31. PMID: 30447606. |
| Carazo-Barrios L, Archidona-Arranz A, Claros-Ruiz A, García-Basterra I, Garzón-Maldonado FJ, Serrano-Castro V, Gutiérrez-Bedmar M, Barbancho MÁ, De la Cruz Cosme C, García-Campos JM, García-Casares N. Correlation between retinal nerve fibre layer thickness and white matter lesions in Alzheimer's disease. Int J Geriatr Psychiatry. 2021 Jun;36(6):935-942. doi: 10.1002/gps.5496. Epub 2021 Feb 1. PMID: 33387372. |
| Zhao A, Fang F, Li B, Chen Y, Qiu Y, Wu Y, Xu W, Deng Y. Visual Abnormalities Associate With Hippocampus in Mild Cognitive Impairment and Early Alzheimer's Disease. Front Aging Neurosci. 2021 Jan 22;12:597491. doi: 10.3389/fnagi.2020.597491. PMID: 33551787; PMCID: PMC7862343. |
| Méndez-Gómez JL, Pelletier A, Rougier MB, Korobelnik JF, Schweitzer C, Delyfer MN, Catheline G, Monfermé S, Dartigues JF, Delcourt C, Helmer C. Association of Retinal Nerve Fiber Layer Thickness With Brain Alterations in the Visual and Limbic Networks in Elderly Adults Without Dementia. JAMA Netw Open. 2018 Nov 2;1(7):e184406. doi: 10.1001/jamanetworkopen.2018.4406. PMID: 30646353; PMCID: PMC6324371. |

Supplemental Table S3 shows an overview of studies that were omitted from the literature overview on associations of neuronal and microvascular structures and function of the neurovascular coupling unit, as quantified by retinal imaging, with cerebral MRI features of structural brain abnormalities in Table 2.

Abbreviations: MRI, magnetic resonance imaging.

Supplemental Table S4 Studies omitted from the literature overview for associations of RNFL thickness or retinal microvascular diameters with cognitive performance or cognitive status

| Study population size N=300-499 |
| --- |
| Méndez-Gómez JL, Rougier MB, Tellouck L, Korobelnik JF, Schweitzer C, Delyfer MN, Amieva H, Dartigues JF, Delcourt C, Helmer C. Peripapillary Retinal Nerve Fiber Layer Thickness and the Evolution of Cognitive Performance in an Elderly Population. Front Neurol. 2017 Mar 21;8:93. doi: 10.3389/fneur.2017.00093. PMID: 28373855; PMCID: PMC5358525. |
| Study population size N<300 |
| Mei X, Qiu C, Zhou Q, Chen Z, Chen Y, Xu Z, Zou C. Changes in retinal multilayer thickness and vascular network of patients with Alzheimer's disease. Biomed Eng Online. 2021 Oct 3;20(1):97. doi: 10.1186/s12938-021-00931-2. PMID: 34602087; PMCID: PMC8489058. |
| Galvin JE, Kleiman MJ, Walker M. Using Optical Coherence Tomography to Screen for Cognitive Impairment and Dementia. J Alzheimers Dis. 2021;84(2):723-736. doi: 10.3233/JAD-210328. PMID: 34569948. |
| Lian TH, Jin Z, Qu YZ, Guo P, Guan HY, Zhang WJ, Ding DY, Li DN, Li LX, Wang XM, Zhang W. The Relationship Between Retinal Nerve Fiber Layer Thickness and Clinical Symptoms of Alzheimer's Disease. Front Aging Neurosci. 2021 Jan 29;12:584244. doi: 10.3389/fnagi.2020.584244. PMID: 33584241; PMCID: PMC7878673. |
| Zhao A, Fang F, Li B, Chen Y, Qiu Y, Wu Y, Xu W, Deng Y. Visual Abnormalities Associate With Hippocampus in Mild Cognitive Impairment and Early Alzheimer's Disease. Front Aging Neurosci. 2021 Jan 22;12:597491. doi: 10.3389/fnagi.2020.597491. PMID: 33551787; PMCID: PMC7862343. |
| Fickweiler W, Wolfson EA, Paniagua SM, Yu MG, Adam A, Bahnam V, Sampani K, Wu IH, Musen G, Aiello LP, Shah H, Sun JK, King GL. Association of Cognitive Function and Retinal Neural and Vascular Structure in Type 1 Diabetes. J Clin Endocrinol Metab. 2021 Mar 25;106(4):1139-1149. doi: 10.1210/clinem/dgaa921. PMID: 33378459; PMCID: PMC7993575. |
| Fang M, Strand K, Zhang J, Totillo M, Chen Q, Signorile JF, Jiang H, Wang J. Characterization of retinal microvasculature and its relations to cognitive function in older people after circuit resistance training. Exp Gerontol. 2020 Dec;142:111114. doi: 10.1016/j.exger.2020.111114. Epub 2020 Oct 22. PMID: 33132156; PMCID: PMC7704902. |
| van de Kreeke JA, Nguyen HT, Konijnenberg E, Tomassen J, den Braber A, Ten Kate M, Yaqub M, van Berckel B, Lammertsma AA, Boomsma DI, Tan HS, Visser PJ, Verbraak FD. Longitudinal retinal layer changes in preclinical Alzheimer's disease. Acta Ophthalmol. 2021 Aug;99(5):538-544. doi: 10.1111/aos.14640. Epub 2020 Oct 18. PMID: 33073531; PMCID: PMC8451744. |
| Santangelo R, Huang SC, Bernasconi MP, Falautano M, Comi G, Magnani G, Leocani L. Neuro-Retina Might Reflect Alzheimer's Disease Stage. J Alzheimers Dis. 2020;77(4):1455-1468. doi: 10.3233/JAD-200043. PMID: 32925026. |
| Mammadova N, Neppl TK, Denburg NL, West Greenlee MH. Reduced Retinal Thickness Predicts Age-Related Changes in Cognitive Function. Front Aging Neurosci. 2020 Mar 24;12:81. doi: 10.3389/fnagi.2020.00081. PMID: 32269521; PMCID: PMC7109392. |
| Szegedi S, Dal-Bianco P, Stögmann E, Traub-Weidinger T, Rainer M, Masching A, Schmidl D, Werkmeister RM, Chua J, Schmetterer L, Garhöfer G. Anatomical and functional changes in the retina in patients with Alzheimer's disease and mild cognitive impairment. Acta Ophthalmol. 2020 Nov;98(7):e914-e921. doi: 10.1111/aos.14419. Epub 2020 Mar 25. PMID: 32212415; PMCID: PMC7687124. |
| Cipollini V, Abdolrahimzadeh S, Troili F, De Carolis A, Calafiore S, Scuderi L, Giubilei F, Scuderi G. Neurocognitive Assessment and Retinal Thickness Alterations in Alzheimer Disease: Is There a Correlation? J Neuroophthalmol. 2020 Sep;40(3):370-377. doi: 10.1097/WNO.0000000000000831. PMID: 31453919. |
| Zhang YS, Onishi AC, Zhou N, Song J, Samra S, Weintraub S, Fawzi AA. Characterization of Inner Retinal Hyperreflective Alterations in Early Cognitive Impairment on Adaptive Optics Scanning Laser Ophthalmoscopy. Invest Ophthalmol Vis Sci. 2019 Aug 1;60(10):3527-3536. doi: 10.1167/iovs.19-27135. PMID: 31412112; PMCID: PMC6694736. |
| Alves C, Jorge L, Canário N, Santiago B, Santana I, Castelhano J, Ambrósio AF, Bernardes R, Castelo-Branco M. Interplay Between Macular Retinal Changes and White Matter Integrity in Early Alzheimer's Disease. J Alzheimers Dis. 2019;70(3):723-732. doi: 10.3233/JAD-190152. PMID: 31282416; PMCID: PMC6700635. |
| Zhang YS, Zhou N, Knoll BM, Samra S, Ward MR, Weintraub S, Fawzi AA. Parafoveal vessel loss and correlation between peripapillary vessel density and cognitive performance in amnestic mild cognitive impairment and early Alzheimer's Disease on optical coherence tomography angiography. PLoS One. 2019 Apr 2;14(4):e0214685. doi: 10.1371/journal.pone.0214685. PMID: 30939178; PMCID: PMC6445433. |
| Jones AR, Robbs CM, Edwards CG, Walk AM, Thompson SV, Reeser GE, Holscher HD, Khan NA. Retinal Morphometric Markers of Crystallized and Fluid Intelligence Among Adults With Overweight and Obesity. Front Psychol. 2018 Dec 21;9:2650. doi: 10.3389/fpsyg.2018.02650. PMID: 30622502; PMCID: PMC6309102. |
| Liu YL, Hsieh YT, Chen TF, Chiou JM, Tsai MK, Chen JH, Chen YC. Retinal ganglion cell-inner plexiform layer thickness is nonlinearly associated with cognitive impairment in the community-dwelling elderly. Alzheimers Dement (Amst). 2018 Nov 12;11:19-27. doi: 10.1016/j.dadm.2018.10.006. PMID: 30581972; PMCID: PMC6297049. |
| Cabrera DeBuc D, Somfai GM, Arthur E, Kostic M, Oropesa S, Mendoza Santiesteban C. Investigating Multimodal Diagnostic Eye Biomarkers of Cognitive Impairment by Measuring Vascular and Neurogenic Changes in the Retina. Front Physiol. 2018 Dec 6;9:1721. doi: 10.3389/fphys.2018.01721. PMID: 30574092; PMCID: PMC6291749. |
| Santos CY, Johnson LN, Sinoff SE, Festa EK, Heindel WC, Snyder PJ. Change in retinal structural anatomy during the preclinical stage of Alzheimer's disease. Alzheimers Dement (Amst). 2018 Feb 7;10:196-209. doi: 10.1016/j.dadm.2018.01.003. PMID: 29780864; PMCID: PMC5956814. |
| Jiang H, Wei Y, Shi Y, Wright CB, Sun X, Gregori G, Zheng F, Vanner EA, Lam BL, Rundek T, Wang J. Altered Macular Microvasculature in Mild Cognitive Impairment and Alzheimer Disease. J Neuroophthalmol. 2018 Sep;38(3):292-298. doi: 10.1097/WNO.0000000000000580. PMID: 29040211; PMCID: PMC5902666. |
| Ryan CM, Klein BEK, Lee KE, Cruickshanks KJ, Klein R. Associations between recent severe hypoglycemia, retinal vessel diameters, and cognition in adults with type 1 diabetes. J Diabetes Complications. 2016 Nov-Dec;30(8):1513-1518. doi: 10.1016/j.jdiacomp.2016.08.010. Epub 2016 Aug 14. PMID: 27601058; PMCID: PMC5050129. |
| Pillai JA, Bermel R, Bonner-Jackson A, Rae-Grant A, Fernandez H, Bena J, Jones SE, Ehlers JP, Leverenz JB. Retinal Nerve Fiber Layer Thinning in Alzheimer's Disease: A Case-Control Study in Comparison to Normal Aging, Parkinson's Disease, and Non-Alzheimer's Dementia. Am J Alzheimers Dis Other Demen. 2016 Aug;31(5):430-6. doi: 10.1177/1533317515628053. Epub 2016 Feb 16. PMID: 26888864. |
| Shi Z, Zhu Y, Wang M, Wu Y, Cao J, Li C, Xie Z, Shen Y. The Utilization of Retinal Nerve Fiber Layer Thickness to Predict Cognitive Deterioration. J Alzheimers Dis. 2016;49(2):399-405. doi: 10.3233/JAD-150438. PMID: 26484909. |
| Tatham AJ, Boer ER, Rosen PN, Della Penna M, Meira-Freitas D, Weinreb RN, Zangwill LM, Medeiros FA. Glaucomatous retinal nerve fiber layer thickness loss is associated with slower reaction times under a divided attention task. Am J Ophthalmol. 2014 Nov;158(5):1008-17. doi: 10.1016/j.ajo.2014.07.028. Epub 2014 Jul 25. PMID: 25068641; PMCID: PMC4515218. |
| Ascaso FJ, Cruz N, Modrego PJ, Lopez-Anton R, Santabárbara J, Pascual LF, Lobo A, Cristóbal JA. Retinal alterations in mild cognitive impairment and Alzheimer's disease: an optical coherence tomography study. J Neurol. 2014 Aug;261(8):1522-30. doi: 10.1007/s00415-014-7374-z. Epub 2014 May 21. PMID: 24846203. |
| Patton N, Pattie A, MacGillivray T, Aslam T, Dhillon B, Gow A, Starr JM, Whalley LJ, Deary IJ. The association between retinal vascular network geometry and cognitive ability in an elderly population. Invest Ophthalmol Vis Sci. 2007 May;48(5):1995-2000. doi: 10.1167/iovs.06-1123. PMID: 17460252. |
| Ong YT, Hilal S, Cheung CY, Xu X, Chen C, Venketasubramanian N, Wong TY, Ikram MK. Retinal vascular fractals and cognitive impairment. Dement Geriatr Cogn Dis Extra. 2014 Aug 27;4(2):305-13. doi: 10.1159/000363286. PMID: 25298774; PMCID: PMC4176466. |
| Ryan CM, Klein BEK, Lee KE, Cruickshanks KJ, Klein R. Associations between recent severe hypoglycemia, retinal vessel diameters, and cognition in adults with type 1 diabetes. J Diabetes Complications. 2016 Nov-Dec;30(8):1513-1518. doi: 10.1016/j.jdiacomp.2016.08.010. Epub 2016 Aug 14. PMID: 27601058; PMCID: PMC5050129 |

Supplemental Table S4 shows an overview of studies that were omitted from the literature overview in Table 2 on associations of RNFL thickness and retinal microvascular diameters with cognitive performance or cognitive status.

Abbreviations: RNFL, retinal nerve fiber layer.

Supplemental Table S5 Studies omitted from the literature overview on OCT-A and cognitive performance or cognitive status

| Study population size N<150 |
| --- |
| O'Bryhim BE, Lin JB, Van Stavern GP, Apte RS. OCT Angiography Findings in Preclinical Alzheimer's Disease: 3-Year Follow-Up. Ophthalmology. 2021 Oct;128(10):1489-1491. doi: 10.1016/j.ophtha.2021.02.016. Epub 2021 Feb 19. PMID: 33610626. |
| Zabel P, Kaluzny JJ, Zabel K, Kaluzna M, Lamkowski A, Jaworski D, Makowski J, Gebska-Toloczko M, Kucharski R. Quantitative assessment of retinal thickness and vessel density using optical coherence tomography angiography in patients with Alzheimer's disease and glaucoma. PLoS One. 2021 Mar 19;16(3):e0248284. doi: 10.1371/journal.pone.0248284. PMID: 33739997; PMCID: PMC7978346. |
| Biscetti L, Lupidi M, Luchetti E, Eusebi P, Gujar R, Vergaro A, Cagini C, Parnetti L. Novel noninvasive biomarkers of prodromal Alzheimer disease: The role of optical coherence tomography and optical coherence tomography-angiography. Eur J Neurol. 2021 Jul;28(7):2185-2191. doi: 10.1111/ene.14871. Epub 2021 Apr 30. PMID: 33852770 |
| Mei X, Qiu C, Zhou Q, Chen Z, Chen Y, Xu Z, Zou C. Changes in retinal multilayer thickness and vascular network of patients with Alzheimer's disease. Biomed Eng Online. 2021 Oct 3;20(1):97. doi: 10.1186/s12938-021-00931-2. PMID: 34602087; PMCID: PMC8489058. |
| Arthur E, Alber J, Thompson LI, Sinoff S, Snyder PJ. OCTA reveals remodeling of the peripheral capillary free zones in normal aging. Sci Rep. 2021 Aug 2;11(1):15593. doi: 10.1038/s41598-021-95230-0. PMID: 34341456; PMCID: PMC8329222. |
| Kwapong WR, Gao Y, Yan Y, Zhang Y, Zhang M, Wu B. Assessment of the outer retina and choroid in white matter lesions participants using swept-source optical coherence tomography. Brain Behav. 2021 Aug;11(8):e2240. doi: 10.1002/brb3.2240. Epub 2021 Jul 21. PMID: 34291589; PMCID: PMC8413737. |
| Li ZB, Lin ZJ, Li N, Yu H, Wu YL, Shen X. Evaluation of retinal and choroidal changes in patients with Alzheimer's type dementia using optical coherence tomography angiography. Int J Ophthalmol. 2021 Jun 18;14(6):860-868. doi: 10.18240/ijo.2021.06.11. PMID: 34150541; PMCID: PMC8165633. |
| Robbins CB, Grewal DS, Stinnett SS, Soundararajan S, Yoon SP, Polascik BW, Liu AJ, Burke JR, Fekrat S. Assessing the Retinal Microvasculature in Individuals With Early and Late-Onset Alzheimer's Disease. Ophthalmic Surg Lasers Imaging Retina. 2021 Jun;52(6):336-344. doi: 10.3928/23258160-20210528-06. Epub 2021 Jun 1. PMID: 34185588. |
| Zhang S, Kwapong WR, Yang T, Liu P, Tuo Q, Cheng Y, Li X, Liu M, Lei P, Wu B. Choriocapillaris Changes Are Correlated With Disease Duration and MoCA Score in Early-Onset Dementia. Front Aging Neurosci. 2021 Apr 13;13:656750. doi: 10.3389/fnagi.2021.656750. PMID: 33927609; PMCID: PMC8076507. |
| Wong MNK, Lai DWL, Chan HH, Lam BY. Neural and Retinal Characteristics in Relation to Working Memory in Older Adults with Mild Cognitive Impairment. Curr Alzheimer Res. 2021;18(3):185-195. doi: 10.2174/1567205018666210608114044. PMID: 34102976. |
| Fang M, Strand K, Zhang J, Totillo M, Signorile JF, Galvin JE, Wang J, Jiang H. Retinal vessel density correlates with cognitive function in older adults. Exp Gerontol. 2021 Sep;152:111433. doi: 10.1016/j.exger.2021.111433. Epub 2021 Jun 6. PMID: 34091000; PMCID: PMC8521640. |
| Montorio D, Criscuolo C, Breve MA, Lanzillo R, Salvatore E, Morra VB, Cennamo G. Radial peripapillary vessel density as early biomarker in preperimetric glaucoma and amnestic mild cognitive impairment. Graefes Arch Clin Exp Ophthalmol. 2022 Jan 22. doi: 10.1007/s00417-022-05561-5. Epub ahead of print. PMID: 35064364 |

Supplemental Table S5 shows an overview of studies that were omitted from the literature overview on OCT-A and cognitive performance or cognitive status in Table 3.

Abbreviations: OCT-A, optical coherence tomography angiography.
